# Pharmacogenetic association of the *NR1H3* promoter variant with antihypertensive response among patients with hypertension: A longitudinal study

**DOI:** 10.3389/fphar.2023.1083134

**Published:** 2023-03-06

**Authors:** Yu Chen, Yuqing Han, Yiyi Wu, Rutai Hui, Yunyun Yang, Yixuan Zhong, Shuyuan Zhang, Weili Zhang

**Affiliations:** ^1^ National Clinical Research Center for Cardiovascular Diseases, State Key Laboratory of Cardiovascular Disease, Fuwai Hospital, Chinese Academy of Medical Sciences and Peking Union Medical College, National Center for Cardiovascular Diseases, Beijing, China; ^2^ The First Affiliated Hospital of Anhui University of Science and Technology, The First People’s Hospital of Huainan City, Huainan, Anhui, China; ^3^ Clinical Laboratory, Xiamen Key Laboratory of Genetic Testing, The First Affiliated Hospital of Xiamen University, Xiamen, Fujian, China; ^4^ Central-China Branch of National Center for Cardiovascular Diseases, Henan Cardiovascular Disease Center, Fuwai Central-China Hospital, Zhengzhou, China

**Keywords:** pharmacogenetics, antihypertensive response, blood pressure, genetic variants, antihypertensive drugs

## Abstract

**Background:** The genetic factors in assessing therapeutic efficacy and predicting antihypertensive drug response are unclear. Therefore, this study aims to identify the associations between variants and antihypertensive drug response.

**Methods:** A longitudinal study including 1837 hypertensive patients was conducted in Northern China and followed up for a median 2.24 years. The associations of 11 candidate variants with blood pressure changes in response to antihypertensive drugs and with the risk of cardiovascular events during the follow-up were examined. The dual-luciferase assay was carried out to assess the effect of genetic variants on gene transcriptional activity.

**Results:** The variant rs11039149A>G in the promoter of nuclear receptor subfamily 1 group H member 3 (*NR1H3*) was associated with the change in systolic blood pressure (ΔSBP) in response to calcium channel blockers (CCBs) monotherapy. Patients carrying rs11039149AG genotype showed a significant increase of systolic blood pressure (SBP) at follow-up compared with AA carriers, and the difference of ΔSBP between AG and AA carriers was 5.94 mm Hg (95%CI: 2.09–9.78, *p* = 0.002). In 1,184 patients with CCBs therapy, SBP levels decreased in AA carriers, but increased in AG carriers, the difference of ΔSBP between AG and AA carriers was 8.04 mm Hg (95%CI: 3.28–12.81, *p* = 0.001). Further analysis in 359 patients with CCBs monotherapy, the difference of ΔSBP between AG and AA carriers was 15.25 mm Hg (95%CI: 6.48–24.02, *p* = 0.001). However, there was no significant difference in ΔSBP between AG and AA carriers with CCBs multitherapy. The rs11039149A>G was not associated with the cardiovascular events incidence during the follow-up. Additionally, transcriptional factor forkhead box C1 (*FOXC1*) bound to the *NR1H3* promoter containing rs11039149A and significantly increased the transcriptional activity, while rs11039149 A to G change led to a loss-of-function and disabled *FOXC1* binding. For the other 10 variants, associations with blood pressure changes or risk of cardiovascular events were not observed.

**Conclusion:** Hypertensive patients with rs11039149AG genotype in the *NR1H3* gene have a significant worse SBP control in response to CCBs monotherapy compared with AA carriers. Our findings suggest that the *NR1H3* gene might act as a promising genetic factor to affect individual sensitivity to antihypertensive drugs.

## 1 Introduction

Hypertension is a major risk factor leading to cardiovascular diseases (Carey et al., 2022). About one-third of Chinese adults suffer from hypertension, while only 29.6% of patients with antihypertensive treatment achieved the blood pressure goal (Lewington et al., 2016). Interindividual genetic variability might explain this disappointing outcome partly. However, the genetic factors in assessing therapeutic efficacy and predicting antihypertensive drug response are unclear.

The blood pressure levels are influenced by environmental and genetic factors (Niu et al., 2021), and genetic factors have shown inheritability from 30% to 50% on blood pressure variation among Chinese and White individuals (Wu et al., 2011; Ehret & Caulfield, 2013; Munroe et al., 2013). Pharmacogenomic studies of hypertension have suggested that genetic variants related to blood pressure elevation showed effects on antihypertensive response in Chinese, Americans, Europeans and Hispanics, etc. (Johnson, 2012; Gong et al., 2015; Iniesta et al., 2019; Citterio et al., 2021; Xiao et al., 2022). Previous genome-wide association studies and candidate gene strategy studies have identified multiple genes and variants related to antihypertensive response. For example, some genes are involved in ion channel function, such as calcium voltage-gated channel subunit alpha1 C (*CACNA1C*) that is found to be associated with antihypertensive response to calcium channel blockers (CCBs) (Beitelshees et al., 2009), and NEDD4 like E3 ubiquitin protein ligase (*NEDD4L*) that is associated with treatment response with beta-blockers or diuretics (Luo et al., 2009; Svensson-Färbom et al., 2011; McDonough et al., 2013). Some genes encode the components of the renin-angiotensin-aldosterone system (RAAS) such as angiotensinogen (*AGT*), angiotensin converting enzyme 2 (*ACE2*) and angiotensin II receptor type 1 (*AGTR1*), which are found to have effects on antihypertensive response to angiotensin converting enzyme inhibitors (ACEIs) or angiotensin receptor blockers (ARBs) (Liljedahl et al., 2004; Fan et al., 2007; Beitelshees et al., 2009; Luo et al., 2009; McDonough et al., 2013; de Denus et al., 2018). However, some genes and variants associated with antihypertensive response still have unclear biological mechanisms.

Genetic variants can be used to explore the efficacy of antihypertensive drugs, but studies have shown that a risk allele for worse response to one drug class might exhibit a beneficial response to another drug class. For example, rs5051 in *AGT* is found to be associated with blood pressure lowering to beta-blockers in 115 Swedish patients with hypertension and left ventricular hypertrophy (Kurland et al., 2004) but with blood pressure lowering to ACEIs in 640 Chinese patients with essential hypertension (Kurland et al., 2004; Yu et al., 2014). Rs5186 in *AGTR1* is related to different therapeutic efficacy to CCBs and ACEIs in 311 White Europeans with hypertension (Benetos et al., 1996) whereas associated with ARBs on systolic blood pressure (SBP) response in 1,049 Chinese patients with hypertension (Jiang et al., 2011). These inconsistent associations may have multiple reasons unrelated to the pharmacology, or due to methodological differences, or led by ethnics of participants. Other variants in *CACNA1C* (Beitelshees et al., 2009), *ACE2* (Fan et al., 2007), *NEDD4L* (Luo et al., 2009; Svensson-Färbom et al., 2011; McDonough et al., 2013), adducin 1 (*ADD1*) (Turner et al., 2003; Vormfelde & Brockmöller, 2012), matrix metallopeptidase 3 (*MMP3*) (Sherva et al., 2011), nuclear receptor subfamily 1 group H member 3 (*NR1H3*) (Price et al., 2011), and protein tyrosine phosphatase receptor type D (*PTPRD*) (Gong et al., 2015) are also shown to have relationship with antihypertensive response or cardiovascular events in response to antihypertensive medications among the Chinese population or other population (including White Europeans, White Americans, Black Americans, Hispanics, etc.), but these results varied between populations and thus should be further explored.

The investigations for genetic variants on antihypertensive response in Chinese population are limited. In this study, a total of 11 variants at 9 genes (*AGT, AGTR1, ADD1, ACE2, CACNA1C, NEDD4L, NR1H3, PTPRD, MMP3*) are chosen and genotyped in a Chinese prospective cohort study including 1837 patients with hypertension, aiming to explore the association of variants with the changes in blood pressure in response to antihypertensive drugs treatment and the risk of cardiovascular events during the follow-up.

## 2 Materials and methods

### 2.1 Study population

This prospective study was conducted in two communities, the Benxi County, Liaoning Province, and Hongxinglong County, Heilongjiang province in the northern region in China. A total of 1953 patients with hypertension (>40 years) were enrolled at two time periods according to the same criteria of inclusion and exclusion, of whom 463 patients from January to November in 2009 and 1,490 patients from January 2012 to December 2014. The definition of hypertension was based on the following criteria: SBP≥140 mm Hg and/or diastolic blood pressure (DBP)≥90 mm Hg, and/or receiving antihypertensive drugs, and/or history of hypertension. The enrolled patients were provided with antihypertensive drugs at free of charge, including CCBs, ARBs, ACEIs, thiazide-type diuretics (hydrochlorothiazide), or beta-blockers, unless intolerance was reported. The drug dosages and types were modulated by doctors according to the patients’ blood pressure levels.

All patients were followed up face-to-face every 2 years by trained investigators, and the last visit was from May 1 to November 30 in 2016. The main endpoint was the composite of stroke, myocardial infarction, coronary revascularization, hospitalization for unstable angina or acute decompensated heart failure, and deaths from cardiovascular causes. Definitions of the endpoints were described in the Supplementary Material.

**FIGURE 1:**
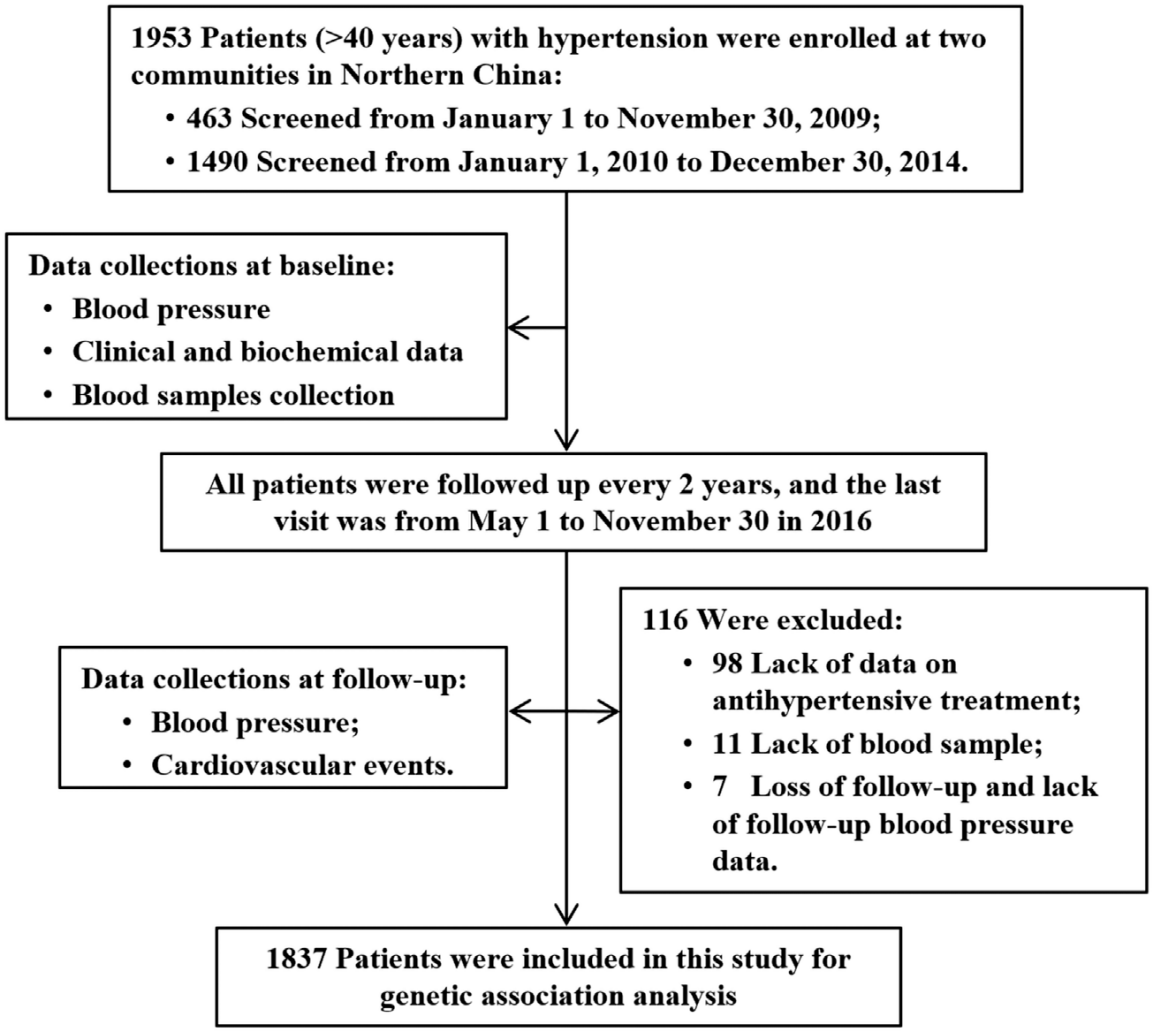
Flowchart of the current study. Cardiovascular events including stroke, myocardial infarction, coronary revascularization, hospitalization for unstable angina or acute decompensated heart failure, and deaths from cardiovascular causes.

Before data assessment, we excluded 98 patients lack of antihypertensive treatment data, 11 patients without genotyping data due to lack of blood samples, and 7 patients who were loss of follow-up due to immigration and lack of follow-up blood pressure. Thus, a total of 1837 patients with complete clinical data and genetic information were included in this study for analyzing the association of variants with blood pressure lowering response and risk of cardiovascular events. The flowchart of study was shown in Figure 1.

### 2.2 Data collection and blood pressure measurements

Baseline demographic characteristics including age, sex, antihypertensive therapy, medical history, and lifestyles (smoking and alcohol status) of enrolled patients were collected through interviews, and a standardized questionnaire was utilized for data collection. Height and weight were measured by skilled nurses, and body mass index was computed as weight (kilograms) divided by the square of height (meters). Blood samples were drawn from the antecubital vein after overnight fast and total cholesterol, low-density lipoprotein cholesterol, high-density lipoprotein cholesterol, triglycerides, fasting blood glucose and serum creatinine were examined by an automatic analyzer (Hitachi 7,060, Tokyo, Japan). All measurements were done at Beijing FuWai Clinical Laboratory and certified by the Centers for Disease Control and Prevention.

Blood pressure was measured by trained nurses using validated oscillometric monitors with suitable-sized arm cuffs (large, medium, or small). All patients were requested to avoid smoke, alcohol, tea, coffee, and exercise for at least 30 min before the blood pressure was measured. The sitting blood pressure was measured 3 times at intervals of 1 min after at least 5 min of rest, the average of the second and third readings was recorded as the blood pressure level. The changes in blood pressure were derived from follow-up blood pressure minus baseline blood pressure. ΔSBP and ΔDBP were used for indicating the changes in SBP and DBP, respectively.

### 2.3 Gene variants selection and genotyping

Eleven genetic variants linked to antihypertensive response were genotyped in this study. These variants were located at 9 genes, including 3 genes involved in the RAAS (*AGT*, *AGTR1*, *ACE2*), 2 genes involved in ion channels (*CACNA1C*, *NEDD4L*), 3 genes associated with vascular functions (*NR1H3*, *PTPRD*, *MMP3*), and *ADD1* gene, which encodes adducin-1 and plays important roles in the stabilization of the membrane cortical cytoskeleton, cell-cell adhesions, and cell signal transduction (Manunta & Bianchi, 2006). Genetic variants were listed in Supplementary Table S1.

Genomic DNA was extracted from the peripheral white blood cell with FlexGen Blood DNA Kit (Cowin Biotech Co., Beijing, China). Genotyping was performed using the Nascent Genotyping system with a custom-by-design 48-Plex SNPscan™ Kit (Genesky Biotechnologies Co., Shanghai, China) which was based on double ligation and multiplex fluorescence polymerase chain reaction (PCR) method. For each variant, two special primers (one for wild allele and the other for mutant allele) and one common primer were designed (Supplementary Table S2). The procedure was as the following: a ligation mixture was first prepared in 20 μl, containing 100 ng DNA sample, 1x ligase buffer, 1U ligase and 1 x primer mix. The ligation reaction was carried out in an ABI2720 thermal cycler under the following cycling program: 95°C for 5 min, 4 cycles of 94°C for 1 min, 58°C for 4 min, 94°C for 2 min, hold at 72°C. Two 48-multiplex fluorescence PCR reactions were performed for each ligation product. The PCR mixture was prepared in 20 μl, containing 1 μl ligation product, 1x primer mix and 1x PCR master mix. The PCR program was described as following: 95°C for 2 min; 9 cycles of 94°C for 20 s, 62°C–0.5°C/cycle for 40 s, and 72°C for 1.5 min; 25 cycles of 94°C for 20 s, 57°C for 40 s, and 72°C for 1.5 min; 68°C for 1 h; and hold at 4°C. PCR products were then detected by capillary electrophoresis on an ABI 3730XL sequencer. Genotyping data were analyzed by the software GeneMapper version 4.1.

The genotyping method used in this study has been validated by single nucleotide extension with the Multiplex SNaPshot Kit (Applied Biosystems Inc., Foster City, CA, USA) in previous studies which have reported >99% concordance rates of validation (Chen et al., 2012; Lu et al., 2021). In our study, Genotyping for eleven variants had completion rates of 99.8%. For quality control, we chose 5% of samples randomly and repeated genotyping, and the concordance rate of repeated samples was 100%. The phenotype status of the patients was masked throughout the genotyping.

### 2.4 Construction of plasmids

Bioinformatic analysis (JASPAR database, http://jaspar.genereg.net/) was used to explore the potential binding sequence of transcriptional factors in relation to the genetic variants in this study. To verify whether rs11039149A>G influences the transcriptional activity of *NR1H3*, 200-bp *NR1H3* promoter fragments containing rs11039149A or rs11039149G allele were amplified by PCR method using the following primers: forward 5′-GGCGGT​ACCCCT​CAG​CAG​CTT​GCC​TCC-3′, reverse 5′- GGCACG​CGTAGT​GGC​TGG​G CTGGGATCAGA-3′, then cloned into the pGL3-promoter luciferase vector (Promega, WI, USA) at KpnI and MluI restriction sites, and two plasmids were generated: pGL3-rs11039149A and pGL3-rs11039149G. Forkhead box C1 (*FOXC1*) gene coding sequence was amplified using the following primers: forward 5′-GCGCG​ATC​GCATG​CAG​GCG​CGC​TAC​TCC-3′, reverse 5′- GGCACGCGTCA AAA​CTT​GCT​ACA​GTC​GTA​G-3′, then cloned into eukaryotic expression vector pENTER (WZ Biosciences, Shandong, China) at AsisI and MluI restriction sites to generate the pENTER-*FOXC1* plasmid. All plasmids were confirmed by DNA sequencing.

### 2.5 Cell culture, transfection, and dual-luciferase assays

HEK293T cells were seeded at a density of 2.0 × 10^4^ cells per well in a 96-well plate, and transfection was performed with lipofectamin3000 (Invitrogen, USA) according to the manufacturer’s protocol. Luciferase vector pGL3-rs11039149A or pGL3-rs11039149G were co-transfected with a transcription factor expression plasmid pENTER-*FOXC1* or pENTER empty vector (each vector 20 ng per well). The *Renilla* luciferase plasmid pRL-TK (Promega, WI, USA) were co-transfected as an internal control (1 ng per well) to standardize the transfection efficiency. The transfected cells were harvested after 36 h, and the luciferase activity was measured using the dual-luciferase reporter assay system (Promega, WI, USA) by a luminometer (TD-20; Turner Designs).

### 2.6 Statistical analysis

Hardy-Weinberg equilibrium for each variant was examined by chi-square test. We used fixed-effect model as the main analysis of genetic association, given that fixed-effect model is more appropriate for exploratory or predictive studies with large sample sizes greater than 500, and it has a smaller median absolute error (4%) of the true marginal effect than random-effect model, as proposed by a simulation study (Dieleman & Templin, 2014). Univariable general linear model was used to explore the confounding variables that might affect the changes in blood pressure, and baseline characteristics of age, sex, body mass index, serum creatine, blood pressure, smoking status, alcohol intake and antihypertensive drugs were found to be significantly associated with the changes in blood pressure (Supplementary Table S3), therefore, these variables were adjusted as fixed effect. In addition, random-effect model was also used to re-analyze the main results, which further adjusted entry time and center as random effect.

In this study, two parallel models were used to give more comprehensive assessments for the genetic association analysis. Generalized linear regression model was used to calculate the means, mean differences and 95% confidence intervals (CIs) of the changes in blood pressure (ΔSBP and ΔDBP) among different genotypes of the variants. Multivariate linear regression model was used to obtain the correlation coefficient of genetic variants with the changes in blood pressure. The effects of antihypertensive treatments and sex on the correlation of variants with the changes in blood pressure were analyzed in further stratification. In addition, we performed epistasis analysis using the PLINK 1.9 (cog-genomics.org) to further explore the effects of interactions of the studied variants on the changes in blood pressure at the follow-up. The *P*-values for interaction were calculated by multiple linear regression models with adjustment for covariates as mentioned above.

Cox proportional-hazards model was used to compute hazards ratios and 95%CIs for the association between variants and the risk of cardiovascular events. Person-years of follow-up began on the date of enrollment until the date of cardiovascular outcomes, death, or the end of follow-up (30 November 2016), whichever came first.

False discovery rate (FDR) derived from the Benjamin & Hochberg method (Smeland et al., 2020) was calculated to correct for multiple comparisons of genetic associations. A two-tailed probability value of ≤0.05 was considered as significant. SPSS Statistics 25.0 (SPSS Inc., Chicago, USA) were used to conduct the analysis.

## 3 Results

### 3.1 Baseline characteristics of patients with hypertension

A total of 1837 patients were included in this study and the baseline characteristics of these patients were shown in Table 1. The mean age of the patients was 63.0 years, and 39.6% were men. The means of baseline SBP and DBP of the patients were 156 mm Hg and 89.5 mm Hg, respectively. The patients received monotherapy or multitherapy of antihypertensive drugs: 64.5% patients had CCBs, 53.2% had ARBs, 12.0% had ACEIs, 22.5% had diuretics, and 20.7% had beta-bockers.

**TABLE 1:** Baseline characteristics of the patients in this study.

Characteristics	All patients (*n* = 1837)
Age, years	63.0 ± 9.6
Men, no. (%)	727 (39.6)
BMI, kg/m^2^	26.3 ± 3.4
SBP, mm Hg	156 ± 22
DBP, mm Hg	89.5 ± 12.1
Lipids, mmol/L	
Total cholesterol	5.54 ± 1.04
Triglycerides	1.62 (1.12–2.34)
HDL-C	1.36 ± 0.31
LDL-C	3.55 ± 0.83
Fasting serum glucose, mmol/L	5.69 (5.24–6.45)
Serum creatinine, μmol/L	76.5 ± 20.4
Cigarette smoking, no. (%)	589 (32.1)
Alcohol intake, no. (%)	581 (31.6)
Medical history, no. (%)	
Coronary heart disease	513 (27.9)
Diabetes	390 (21.2)
Stroke	349 (19.0)
Antihypertensive drugs, no. (%)	
Calcium channel blockers	1,184 (64.5)
Angiotensin receptor blockers	977 (53.2)
ACE inhibitors	221 (12.0)
Diuretics	414 (22.5)
Beta-blockers	380 (20.7)

Abbreviations: BMI, body mass index; SBP, systolic blood pressure; DBP, diastolic blood pressure; HDL-C, high-density lipoprotein cholesterol; LDL-C, low-density lipoprotein cholesterol; ACE, angiotensin converting enzyme.

Values were presented as mean ± standard deviation, number (percentage), or median (interquartile range).

### 3.2 *NR1H3* gene variant rs11039149A>G was associated with the change in SBP

The frequencies of 11 studied variants were shown in Supplementary Table S4 and the frequencies distribution of all variants did not deviate significantly from Hardy-Weinberg equilibrium (all *p* > 0.05). Means, mean differences and 95%CIs of blood pressure changes between genotypes were calculated by generalized linear regression model with adjustment for age, sex, body mass index, serum creatine, blood pressure, smoking status, alcohol intake and antihypertensive drugs. The results showed that *NR1H3* gene variant rs11039149A>G was associated with ∆SBP. In this studied population, the minor allele frequency of rs11039149G was 3.0%, and the genotype frequencies of rs11039149 were respectively as AA (94.0%) and AG (6.0%). ∆SBP was 2.95 mm Hg (95%CI: −1.08 to 6.98) for the *NR1H3* variant rs11039149AG carriers and −2.99 mm Hg (95%CI: −4.71 to −1.26) for the AA carriers; ∆DBP was −0.56 mm Hg (95%CI: −2.68 to 1.57) for the AG carriers and −2.66 mm Hg (95%CI: −3.57 to −1.57) for the AA carriers. The difference of ∆SBP between AG and AA carriers was 5.94 mm Hg (95%CI: 2.09 to 9.78, *p* = 0.002, FDR = 0.02), but no significant difference of ∆DBP was found between the AG and AA carriers after multiple comparison correction by the Benjamin & Hochberg method (FDR = 0.44) (Table 2).

**TABLE 2:** Effects of *NR1H3* variant rs11039149A>G on changes in blood pressure.

Changes in BP	Genotype	Mean (95%CI)^a^ of changes in BP, mm Hg	Mean difference (95%CI)^a^ of changes in BP, mm Hg	*P* ^a^	FDR^b^
*NR1H3* rs11039149A>G					
ΔSBP, mm Hg	AA (*n* = 1726)	−2.99 (−4.71, −1.26)	5.94 (2.09, 9.78)	0.002	0.02
	AG (*n* = 111)	2.95 (−1.08, 6.98)			
ΔDBP, mm Hg	AA (*n* = 1726)	−2.66 (−3.57, −1.75)	2.11 (0.08, 4.13)	0.04	0.44
	AG (*n* = 111)	−0.56 (−2.68, 1.57)			

Abbreviations: *NR1H3*, nuclear receptor subfamily 1 group H member 3; BP, blood pressure; ΔSBP, changes in systolic blood pressure; ΔDBP, changes in diastolic blood pressure; CI, confidence interval; FDR, false discovery rate.

^a^ Means, mean differences, 95%CIs and *P*-values were calculated by generalized linear regression model with adjustment for baseline characteristics including age, sex, body mass index, serum creatine, blood pressure, smoking status, alcohol intake, and antihypertensive drugs. Mean differences were calculated between AG and AA genotypes.

^b^ FDRs were calculated by the Benjamin & Hochberg method.

For the *NR1H3* variant rs11039149A>G, when stratified by sex, the difference of ∆SBP between AG and AA carriers was observed in both men and women (Supplementary Table S5). For the other 10 variants, the associations with blood pressure changes were not observed in either men, women or the whole patients (Supplementary Tables S5, S6).

The correlation coefficients between variants and blood pressure changes were further assessed by multiple linear regression model. The coefficient beta was 5.77 (*p* = 0.003, FDR = 0.03) for ∆SBP and 2.08 (*p* = 0.04, FDR = 0.46) for ∆DBP, respectively (Supplementary Table S7). We also performed epistasis analysis to further explore the effects of interactions between rs11039149 and other 10 studied variants on blood pressure changes at the follow-up. No significant epistasis was found to affect blood pressure changes (all *P* for interaction>0.05) (Supplementary Table S8). Considering the potential effects of entry time and center, we further adjusted these two covariates as random effect in multiple linear mixing model. The results showed that rs11039149AG genotype was significantly associated with SBP increase during the follow-up (*p* = 0.01), which was consistent with the fixed-effect model (Supplementary Table S9).

We compared baseline characteristics of the patients with two genotypes of rs11039149, showing that patients with AG genotype had a lower body mass index and a higher level of serum creatinine than those with AA genotype (Supplementary Table S10). In addition, patients with AG genotype had a higher level of SBP at the follow-up than those with AA genotype. There were no significant differences in other characteristics including age, sex, blood lipid, fasting serum glucose, baseline blood pressure, smoking status, alcohol intake, the usage of antihypertensive drugs and medical history of cardiovascular diseases.

### 3.3 *NR1H3* gene variant rs11039149A>G was associated with SBP response to CCBs

The association of *NR1H3* variant rs11039149A>G with ∆SBP differed in antihypertensive therapy. In 1,184 patients with CCBs therapy, SBP levels decreased in AA carriers (ΔSBP: −2.49 mm Hg, 95%CI: −4.45 to −0.53), but increased in AG carriers (ΔSBP: 5.55 mm Hg, 95%CI: 0.64 to 10.64), the difference of ΔSBP between AG and AA carriers was 8.04 mm Hg (95%CI: 3.28 to 12.81, *p* = 0.001, FDR = 0.01) (Table 3). However, there was no significant difference in ΔSBP between AG and AA carriers without CCBs therapy (*p* = 0.41). As for ARBs, ACEIs, diuretics and beta-blockers, the therapies of these drugs did not significantly affect the association between the *NR1H3* variant rs11039149A>G and blood pressure changes (all *p* > 0.05) (Supplementary Table S11).

**TABLE 3:** Effects of *NR1H3* variant rs11039149A>G on SBP response to CCBs therapy.

Genotype	Mean (95%CI)^a^ of ΔSBP, mm Hg	Mean difference (95%CI)^a^ of ΔSBP, mm Hg	*P* ^a^
*NR1H3* rs11039149A>G			
CCBs therapy (*n* = 1,184)			
AA (*n* = 1,112)	−2.49 (−4.45, −0.53)	8.04 (3.28, 12.81)	0.001
AG (n = 72)	5.55 (0.64, 10.46)		
CCBs monotherapy (*n* = 359)			
AA (n = 335)	−0.96 (−6.89, 4.96)	15.25 (6.48, 24.02)	0.001
AG (n = 24)	14.28 (4.18, 24.38)		
CCBs multitherapy (*n* = 825)			
AA (*n* = 777)	−3.30 (−5.73, −0.88)	4.51 (−1.69, 10.71)	0.15
AG (*n* = 48)	1.21 (−5.14, 7.55)		
non CCBs therapy (*n* = 653)			
AA (*n* = 614)	−3.39 (−5.47, −1.31)	2.05 (−4.42, 8.52)	0.41
AG (*n* = 39)	−1.34 (−7.78, 5.10)		

Abbreviations: *NR1H3*, nuclear receptor subfamily 1 group H member 3; SBP, systolic blood pressure; CCBs, calcium channel blockers; CI, confidence interval; ΔSBP, changes in systolic blood pressure.

^a^ Means, mean differences, 95%CIs and *P*-values were calculated by generalized linear regression model with adjustment for baseline characteristics of age, sex, body mass index, serum creatine, blood pressure, smoking status, alcohol intake and antihypertensive drugs including ARBs, ACEIs, diuretics and beta-blockers (expect for CCBs monotherapy). Mean differences were calculated between AG and AA genotypes.

Among the 1,184 patients with CCBs therapy, 359 (30.3%) patients had CCBs monotherapy and 825 (69.7%) patients had multitherapy. Considering the potential effect of multitherapy on the association of the genotypes with CCBs therapy, we further assessed the differences of ΔSBP in patients with or without rs11039149G allele when receiving CCBs monotherapy and multitherapy. In patients with CCBs monotherapy, the difference of ΔSBP between patients with AG and AA genotype was 15.25 mm Hg (95%CI: 6.48 to 24.02, *p* = 0.001). However, in patients with CCBs multitherapy, there was no significant difference in ΔSBP between the patients with two genotypes (*p* = 0.15). Compared with the patients receiving CCBs multitherapy, the difference in ∆SBP was more significant in patients with CCBs monotherapy (*P* genotype × CCBs therapy interaction = 0.002) (Table 3).

Irregular drug taking (defined as the change in types of drugs, and/or the increase/decrease in the dosage, and/or stop the use of drugs without doctors’ prescription) and drug regimen change (defined as the change in types of drugs during the follow-up) may also affect the genetic association. Therefore, after 100 patients with irregular drug taking or 531 patients with drug regimen change were excluded, we further performed the sensitivity analysis. The results showed that the association of rs11039149 with SBP in response to CCBs still remained. (Supplementary Tables S12, S13).

With the use of multiple linear mixing model, entry time and center were further adjusted as random effect. The results showed that rs11039149A>G was significantly associated with SBP response to CCBs monotherapy (*P*_genotype × CCBs therapy interaction_ = 0.004), which was consistent with the fixed-effect model (Supplementary Table S14).

### 3.4 No variants were associated with the risk of cardiovascular events

All patients in this study were followed up for a median 2.24 years, and 159 events were recorded. There were no significant associations between the 11 variants and the risk of cardiovascular events (all *p* > 0.05, Supplementary Table S15).

### 3.5 *NR1H3* variant rs11039149G allele disabled the transcriptional factor *FOXC1* binding

**FIGURE 2:**
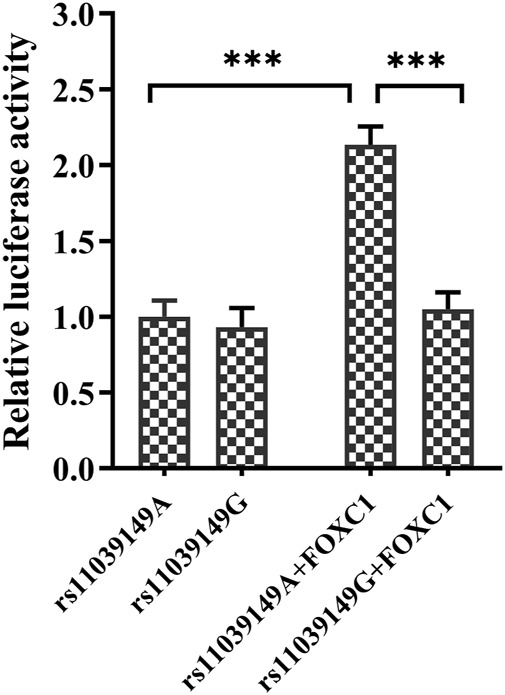
Transcription activity analysis of the *NR1H3* promoter containing rs11039149A and rs11039149G alleles in HEK293T. After co-transfected with the *FOXC1* gene, the wild pGL3-rs11039149A had a significantly higher relative luciferase activity by 2-fold than the mutant pGL3-rs11039149G (***p < 0.001). The pGL3-rs11039149A and pGL3-rs11039149G luciferase reporter vectors carried the *NR1H3* gene promoter fragment containing rs11039149A or rs11039149G allele, respectively. Firefly luciferase activity was expressed relative to pGL3-rs11039149A co-transfected with pENTER empty plasmid. Renilla luciferase activity encoded by the co-transfected control plasmid pRL-TK to standardize the transfection efficiency. Data are presented as mean ± standard error (SE) from three independent experiments.

Bioinformatic analysis (JASPAR database, http://jaspar.genereg.net/) showed that the variant rs11039149 A to G change at the *NR1H3* promoter region could affect the binding ability with transcriptional factor *FOXC1*, which changed the transcription activity of *NR1H3* gene. Therefore, pENTER-*FOXC1* and pGL3-promoter luciferase vectors pGL3-rs11039149A and pGL3-rs11039149G were constructed, and the luciferase activity representative of *NR1H3* promoter activity was measured by the dual-luciferase assay carried out in HEK293T. The relative luciferase activity had no significant difference between the pGL3-rs11039149A and pGL3-rs11039149G when without any stimulation of transcriptional factors. As for the wild pGL3-rs11039149A having potential binding sequence with the *FOXC1*, its relative luciferase activity significantly increased by 2.1-fold comparing the presence of *FOXC1* with the absence of *FOXC1* (*p* < 0.001). After co-transfected with the *FOXC1* gene, the wild pGL3-rs11039149A had a significantly higher relative luciferase activity by 2-fold than the mutant pGL3-rs11039149G (*p* < 0.001) (Figure 2). These results suggested that *FOXC1* could promote the transcriptional activity of *NR1H3 via* binding to the wild rs11039149A allele, while the mutant rs11039149G allele disabled the *FOXC1* binding and had no change in transcriptional activity.

## 4 Discussion

In this longitudinal study, a total of 11 variants at 9 genes (*AGT, AGTR1, ADD1, ACE2, CACNA1C, NEDD4L, NR1H3, PTPRD, MMP3*) were analyzed in 1837 Chinese patients with hypertension. Our data first provided evidence in Chinese patients with hypertension that *NR1H3* promoter variant rs11039149A>G was related to the change in SBP and the SBP response to CCBs monotherapy. However, no significant association was found between *NR1H3* variant rs11039149A>G and the risk of cardiovascular events.

The genetic association analysis showed that SBP levels decreased in patients with rs11039149AA genotype, but increased in patients with AG genotype. In addition, bioinformatics analysis and dual luciferase assay showed that *FOXC1* could bind to rs11039149A and promote the transcriptional activity of *NR1H3*. The rs11039149A to G change disabled *FOXC1* binding with *NR1H3*. *FOXC1* gene encodes fork head protein C1, acting as a transcription factor binding to gene promoter sequence and playing roles in the early development of the heart and blood vessels.

*NR1H3* gene encodes liver X receptor alpha (LXRA), one of the key transcription factors for cholesterol efflux and inflammatory gene responses in macrophages, and thus takes part in the process of atherosclerosis (Che et al., 2021; Savla et al., 2022). LXRA can bind to the promoter region of *renin* gene to regulate *renin* transcription (Morello et al., 2005), and activation of LXRA is reported to suppress the RAAS activation (Kuipers et al., 2010). Animal studies have shown that the LXR agonist GW3965 can reduce blood pressure in rats and have beneficial effects on vascular function (Han et al., 2018; Bal et al., 2019). Supporting, we found that rs11039149G allele is a loss-of-function allele that could disable the *FOXC1* binding, which may partly explain the effects of rs11039149G allele on blood pressure changes. In addition, it has been reported that LXR deficiency can lead to atherosclerosis with increased monocyte entry, foam cell formation, and plaque inflammation (Endo-Umeda et al., 2022). A case-control study in the INVEST-GENES cohort showed that rs11039149G in *NR1H3* is the protective allele for cardiovascular outcomes in White Americans and Hispanics (Price et al., 2011), while it was not found in our study. One reason may be the difference in ethnics, and another reason may be related to multiple complex factors on atherosclerosis and cardiovascular diseases.

*NR1H3* gene variant rs11039149A>G was significantly associated with ∆SBP but not with ∆DBP after multiple comparison correction by the Benjamin & Hochberg method. There are several potential explanations. First, the heritability for blood pressure is shown as 30–50% (Ehret & Caulfield, 2013; Munroe et al., 2013), and SBP has a higher heritability (46%) than that of DBP (30%) in Chinese population (Wu et al., 2011), indicating that SBP may be more susceptible to genetic factors. Second, *NR1H3* regulates lipid homeostasis, inflammation and affects the process of arteriosclerosis (Jarvis et al., 2019), and moreover, atherosclerosis has a greater effect on the increase of SBP (Smulyan et al., 2016). Third, animal studies have shown that activation of *NR1H3* can significantly reduce SBP in rats and mice (Negishi et al., 2018; Bal et al., 2019), and the possible mechanism may be that *NR1H3* could regulate blood pressure through the RAAS (Joseph et al., 2002), which supported the results of the present study to some extent.

Another finding of this study showed that *NR1H3* variant rs11039149G was associated with SBP increase in patients with CCBs monotherapy. CCBs inhibit Ca^2+^ influx through L-type calcium channels in myocardium cells and vascular smooth muscle cell (VSMCs), thus relax vasoconstriction and reduce blood pressure (Laurent, 2017). Clunn et al. found that VSMCs transformation from a differentiated phenotype to a synthetic or dedifferentiated phenotype is associated with loss-of-function of L-type calcium channels and hence loss of potential responsiveness to CCBs (Clunn et al., 2010). The process of VSMCs phenotype switching is associated with lipid metabolism and inflammatory response (Shi et al., 2020). Davies et al. found that the direct activation of LXRs in VSMCs up-regulated FASE, SREBP1c, and SCD-1, thus promotes TG accumulation *via de novo* FA synthesis and SCD-1-mediated Δ^9^ desaturation (Davies et al., 2005). These studies indicate that *NR1H3* gene may play a role in the loss of CCBs effect. In addition, we found that compared with patients receiving CCBs monotherapy, patients receiving CCBs multitherapy had a better blood pressure control. The results showed that the worse SBP control of CCBs in rs11039149AG carriers could be alleviated by combined use with other antihypertensive drugs, which supports the previous studies that combination therapy was more likely to achieve blood pressure goals (Ma et al., 2015; An et al., 2021).

One advantage of the present study was that all patients are of Han ethnicity, which avoided the possibility of spurious association caused by population stratification. In addition, the study was a community-based study and all of the patients were recruited from the same geographic area, which minimized the influences of various lifestyles and environments. Several limitations of the present study need to be mentioned. First, drug adherence was not evaluated in this study, but the questionnaires were used to investigate whether the patients took drugs regularly according to the doctor’s prescription during the follow-up. After excluding 100 patients with irregular drug taking, we performed the sensitivity analysis and obtained a consistent result. Second, the adverse drug reactions were not collected in the database, and thus it was unable to assess the associations of genetic variants with the occurrence of adverse reactions or some grades of adverse reactions. However, in this study, the enrolled patients were provided with antihypertensive drugs (including CCBs, ARBs, ACEIs, thiazide-type diuretics, or beta-blockers, unless intolerance was reported), all of which are proved to be safe and most widely used antihypertensive drug types in China. Due to possible adverse effects, the study could not exclude some bias with regarding to the association of *NR1H3* variant rs11039149A>G with blood pressure changes in response to CCBs. Third, the efficiency of CCBs was influenced by pharmacodynamic effects directly and correlated with its plasma concentration (Taburet et al., 1983), and the genetic variants may have effects on pharmacokinetics and pharmacodynamics (Guo et al., 2015; Wang et al., 2015). However, considering the long-term follow-up and large patient population in this study, the pharmacokinetic assessment of antihypertensive drugs was not included due to infeasibilities. The multiple-effects of genetic variants on antihypertensive drugs should be evaluated precisely in the future. Fourth, the data of antihypertensive drug dosage of each patient was not collected, and thus was unable to investigate the association in a dose-dependent manner. Given that the difference in drug dosages might affect the genetic association of *NR1H3* variant rs11039149A>G with the changes in blood pressure in CCBs, future studies are needed to explore the genetic effects in a dose-dependent manner. Moreover, the results of the present study need to be strengthened by replication in another population, and need to be validated in other races to generalize to a larger population beyond the Han patients.

In conclusion, the key finding of the study is that hypertensive patients with rs11039149AG genotype in the *NR1H3* gene have a significantly worse SBP control in response to the monotherapy of CCBs, compared with patients with the wild-type rs11039149AA genotype. The underlying biological mechanisms may be related to the disabled binding ability of transcription factor *FOXC1* to rs11039149 caused by A to G change. Our findings support that the *NR1H3* gene might act as a promising genetic factor to affect individual sensitivity to antihypertensive drugs. Clinical trials in pharmacogenetics will be helpful to investigate genetically determined variations in response to drugs and thus benefit the individual treatment strategies.

## Data Availability

The original contributions presented in the study are included in the article/Supplementary Material, further inquiries can be directed to the corresponding author.
