# Supplementary material for: Pharmacogenetic association of the NR1H3 promoter variant with antihypertensive response among patients with hypertension: A longitudinal study
Source: Front Pharmacol. 2023 Mar 6;14:1083134. doi: 10.3389/fphar.2023.1083134 (PMC10025344; doi:10.3389/fphar.2023.1083134)
Supplement: Supplementary file 1 [file DataSheet1.docx]

Supplementary Material

Pharmacogenetic association of the *NR1H3* promoter variant with antihypertensive response among patients with hypertension: a longitudinal study

Yu Chen^†^, Yuqing Han^†^, Yiyi Wu, Rutai Hui, Yunyun Yang, Yixuan Zhong, Shuyuan Zhang, Weili Zhang^*^

^†^Yu Chen and Yuqing Han contributed equally to this study.

*** Correspondence:** Weili Zhang: [zhangweili@fuwaihospital.org](mailto:zhangweili@fuwaihospital.org)

**1 Supplementary Methods**

**1.1 Definition of the studied outcomes**

The main end-points included the total cardiovascular diseases (defined as nonfatal myocardial infraction [MI] or fatal coronary heart disease [CHD], and stroke) and deaths from cardiovascular causes.

The diagnosis of myocardial infraction was confirmed if met the criteria of the World Health Organization of the symptoms such as retrosternal pain last for at least 30 minutes and not relieve after taking nitroglycerine, and either electrocardiographic abnormal observed or elevated cardiac enzyme levels (Tunstall et al., 1994). Fatal CHD was recognized to have occurred these situations: fatal MI confirmed by hospital records; CHD or MI as the main cause of death on the death certificate (Theisen et al., 2012). Stroke was diagnosed according to the WHO criteria definition as “the sudden onset of focal (or global) deficit of cerebral function lasting more than 24 hours (except interrupted by surgery or death)” confirmed by a brain computed tomography or magnetic resonance (World Health Organization, 1989).

Deaths were reported by family members, work associates, and/or obtained from death certificates and medical records. They were coded according to ICD-9, and ICD-9 codes 390-459 were classified as cardiovascular deaths.

**1.2 Genotyping of studied genetic variants**

Genomic DNA was isolated from the peripheral white blood cell using Flaxen Blood DNA Kit (Cowing Biotech Co., Beijing, China). Variants were genotyped using the Nascent Genotyping system (Gene sky Biotechnologies Co., Shanghai, China). The SNP genotyping work was performed using a custom-by-design 1x48-Plex Nascent kit (Cat#: G0104; Gene sky Biotechnologies Inc., Shanghai, China), which was based on double ligation and multiplex fluorescence PCR. A random 5% of the samples were repeated to validate genotyping procedures and the concordance rate of repeated samples was 100%. For each variant, two allelic probes (one for wild allele and the other for mutant allele) and one common probe were designed (Supplementary Table S2). The procedure was as the following: a ligation mixture was first prepared in 20 all, containing 100 ng DNA sample, 1x ligase buffer, 1U ligase and 1 x primer mix. The ligation reaction was done on an ABI 2720 thermal cycler under the following cycling program: 95 °C for 5 min, 4 cycles of 94 °C for 1 min, 58 °C for 4 min, 94 °C for 2 min, hold at 72 °C. Two 48-multiplex fluorescence PCR reactions were then performed for each ligation product. The PCR mixture was prepared in 20 μl, containing 1 μl ligation product, 1x primer mix and 1x PCR master mix. The PCR program was described as following: 95 °C for 2 min; 9 cycles of 94 °C for 20 s, 62 °C–0.5 °C/cycle for 40 s, and 72 °C for 1.5 min; 25 cycles of 94 °C for 20 s, 57 °C for 40 s, and 72 °C for 1.5 min; 68 °C for 1 h; and hold at 4 °C. PCR products were then detected by capillary electrophoresis on an ABI 3730XL sequencer. Genotyping data were analyzed by the software GeneMapper version 4.1. The genotyping accuracy has been validated by single nucleotide extension using the Multiplex SNaPshot Kit (Applied Biosystems Inc., Foster City, CA, USA) in previous studies (Chen et al., 2012; Lu et al., 2021), and the concordance rates of validation were more than 99%.

**References:**

Tunstall-Pedoe, H., Kuulasmaa, K., Amouyel, P., Arveiler, D., Rajakangas, A. M., and Pajak, A. (1994). Myocardial infarction and coronary deaths in the world health organization monica project. Registration procedures, event rates, and case-fatality rates in 38 populations from 21 countries in four continents. *Circulation*, 90, 583–612. doi: 10.1161/01.cir.90.1.583

Thygesen, K., Alpert, J. S., Jaffe, A. S., Simoons, M. L., Chaitman, B. R., and White, H. D. (2012). Third universal definition of myocardial infarction. *Glob Heart*, 7, 275-295. doi: 10.1016/j.gheart.2012.08.001

World Health Organization. (1989). Recommendations on stroke prevention, diagnosis, and therapy. Report of the WHO task force on stroke and other cerebrovascular disorders. *Stroke*, 20, 1407–1431. doi: 10.1161/01.str.20.10.1407

Chen, X., Li, S., Yang, Y., Yang, X., Liu, Y., Liu, Y., et al. (2012). Genome-wide association study validation identifies novel loci for atherosclerotic cardiovascular disease. *J Thromb Haemost*, 10, 1508-14. doi: 10.1111/j.1538-7836.2012.04815.x

Lu, C., Zhang, Y., Qin, Y., Xu, Q., Zhou, R., Cui, Y., et al. (2021). Human X chromosome exome sequencing identifies BCORL1 as contributor to spermatogenesis. *J Med Genet*, 58, 56-65. doi: 10.1136/jmedgenet-2019-106598

**2 Supplementary Tables**

Supplementary Table S1. The list of studied genetic variants.

Supplementary Table S2. The primers for genotyping of genetic variants.

Supplementary Table S3. Association of variables with the changes in blood pressure.

Supplementary Table S4. Genotype and allele frequencies of 11 variants in all patients.

Supplementary Table S5. Association of genetic variants with the changes in blood pressure in sex stratification.

Supplementary Table S6. Association of genetic variants with the changes in blood pressure calculated by generalized linear regression model.

Supplementary Table S7. Association of genetic variants with the changes in blood pressure calculated by multivariable linear regression model.

Supplementary Table S8. Association of two-variants interactions between *NR1H3* variant rs11039149 A>G and other variants with the changes in blood pressure.

Supplementary Table S9. Association of *NR1H3* variant rs11039149 A>G with the changes in blood pressure calculated by multiple linear mixing model.

Supplementary Table S10. Baseline characteristics of patients of *NR1H3* variant rs11039149 AA and AG genotypes.

Supplementary Table S11. Association of genetic variants with the blood pressure response to antihypertensive drugs therapy.

Supplementary Table S12. Sensitivity analysis on association of *NR1H3* variant rs11039149 A>G with SBP response to CCBs therapy after excluding 100 patients with irregular drug taking (n=1737).

Supplementary Table S13. Sensitivity analysis on association of *NR1H3* variant rs11039149 A>G with SBP response to CCBs therapy after excluding 531 patients with change in drug regimen (n=1306).

Supplementary Table S14. Association of *NR1H3* variant rs11039149 A>G with SBP response to CCBs therapy calculated by multiple linear mixing model.

Supplementary Table S15. Survival analysis between variants and cardiovascular events.

**Supplementary Table S1. The list of studied genetic variants.**

| **Gene** | **Variant ID** | **Chromosome** | **Location* (bp)** | **Major allele** | **Minor allele** | **HCB MAF** |
| --- | --- | --- | --- | --- | --- | --- |
| *AGT* | rs699 | 1 | 230710048 | G | A | 0.19 |
|  | rs5051 | 1 | 230714126 | T | C | 0.19 |
| *AGTR1* | rs5186 | 3 | 148742201 | A | C | 0.06 |
| *ADD1* | rs4961 | 4 | 2904980 | G | T | 0.46 |
| *PTPRD* | rs4742610 | 9 | 9687487 | C | T | 0.38 |
| *NR1H3* | rs11039149 | 11 | 47255124 | A | G | 0.05 |
| *MMP3* | rs32025058 | 11 | 102845217 | A | del | 0.15 |
| *CACNA1C* | rs1051375 | 12 | 2679713 | A | G | 0.37 |
| *NEDD4L* | rs4149601 | 18 | 58149559 | G | A | 0.19 |
|  | rs292449 | 18 | 58227849 | C | G | 0.19 |
| *ACE2* | rs2106809 | X | 15599938 | G | A | 0.48 |

Abbreviations: HCB MAF, minor allele frequencies of Han Chinese population based on the 1,000 Genomes Project; *AGT*, angiotensinogen; *AGTR1*, angiotensin II receptor type 1; *ADD1*, adducin 1; *PTPRD*, protein tyrosine phosphatase receptor type D; *NR1H3*, nuclear receptor subfamily 1 group H member 3; *MMP3*, matrix metallopeptidase 3; *CACNA1C*, calcium voltage-gated channel subunit alpha1 C; *NEDD4L*, NEDD4 like E3 ubiquitin protein ligase; *ACE2*, angiotensin converting enzyme 2.

*Data based on Genome Reference Consortium Human Build 38 (GRCh38).

**Supplementary Table S2. The primers for genotyping of genetic variants.**

| **Variants** | **Allele sequence special primer 1*** | **Allele sequence especial primer 2^†^** | **Allele universal primer** |
| --- | --- | --- | --- |
| *AGT* rs699 G>A | GCTGTCCACACTGGCTCAC**A** | GCTGTCCACACTGGCTCAC**G** | TCAGGGAGCAGCCAGTCTTC |
| *AGT* rs5051 T>C | CAACGGCAGCTTCTTCCAC**C** | CAACGGCAGCTTCTTCCAC**T** | GGCCGGGTCACGAKGCCCTA |
| *AGTR1* rs5186 A>C | GCAGCACTTCACTACCAAATGATC**C** | GCAGCACTTCACTACCAAATGACC**A** | TTAGCTACTTTTCAGAATTGAAGGA |
| *ADD1* rs4961 G>T | GCGACGAAGCTTCCGAGGCA**T** | GCGACGAAGCTTCCGAGGTA**G** | GGCAGAATGGAAGCAGTCCC |
| *PTPRD*  rs4742610 C>T | CCAAATGTCAGACACCAGTTATGTTTTGT**T** | CCAAATGTCAGACACCAGTTATGTTTTGT**C** | TAGTCCTCAGGAAACTGAAAAATAT |
| *NR1H3* rs11039149 A>G | GCAGCCAGGGCTGGTGGTC**C** | GCAGCCAGGGCTGGTGGTC**T** | ACAGGGGAGAGGCTGAGAACC |
| *MMP3* rs32025058 A>del | TCTCCATTCCTTTGATGGGTG**G** | CTCCATTCCTTTGATGGGGTG**A** | AAAAACCATGTCTTGTCCTGATTGA |
| *CACNA1C* rs1051375 A>G | CCGCCGGCTACCCCAGCTC**G** | CCGCCGGCTACCCCAGCTC**A** | GTCAGCACTGTGGAGGGCCA |
| *NEDD4L* rs4149601 G>A | TGAGACGTCTCGCATTTGAGAA**A** | TGAGACGTCTCGCATTTGAGGA**G** | GTAACACTCGGTAAGACTTTGCTTGG |
| *NEDD4L* rs292449 C>G | TCAGCTTCTGCTCCATTGCTGAT**G** | TCAGCTTCTGCTCCATTGCTGAT**C** | AACTTTTTTTTGGTCGTGACATCAGA |
| *ACE2* rs2106809 G>A | AGCTGCTGATGTAGAAGTGTGGAGATG**T** | AGCTGCTGATGTAGAAGTGTGGAGACG**C** | CCATCAGATAGAGATATGGAAAAAAAAGCT |

* Allele sequence special primer 1 were designed for major alleles.

† Allele sequence special primer 2 were designed for minor alleles.

The full names of the genes are the same as mentioned in the footnote of Supplementary Table S1.

**Supplementary Table S3. Association of variables with the changes in blood pressure.**

| **Variables** | **ΔSBP, mm Hg** | |  | **ΔDBP, mm Hg** | |
| --- | --- | --- | --- | --- | --- |
|  | **R*** | ***P**** |  | **R*** | ***P**** |
| Age, years | -0.001 | 0.96 |  | 0.06 | 0.01 |
| Sex, no | 0.02 | 0.39 |  | 0.06 | 0.01 |
| BMI, kg/m^2^ | -0.002 | 0.93 |  | -0.10 | <0.001 |
| SBP, mm Hg | -0.58 | <0.001 |  | -0.24 | <0.001 |
| DBP, mm Hg | -0.24 | <0.001 |  | -0.54 | <0.001 |
| Lipids, mmol/L |  |  |  |  |  |
| Total cholesterol | -0.03 | 0.27 |  | 0.02 | 0.38 |
| Triglycerides | 0.03 | 0.27 |  | -0.03 | 0.19 |
| HDL-C | 0.004 | 0.85 |  | -0.01 | 0.55 |
| LDL-C | -0.03 | 0.25 |  | 0.02 | 0.33 |
| Fasting serum glucose, mmol/L | -0.04 | 0.10 |  | -0.01 | 0.66 |
| Serum creatinine, μmol/L | 0.14 | <0.001 |  | -0.10 | <0.001 |
| Cigarette smoking, no. (%) | 0.17 | <0.001 |  | -0.12 | <0.001 |
| Alcohol intake, no. (%) | 0.18 | <0.001 |  | -0.12 | <0.001 |
| Medical history, no. (%) |  |  |  |  |  |
| Coronary heart disease | -0.04 | 0.07 |  | -0.01 | 0.56 |
| Diabetes | -0.05 | 0.10 |  | -0.002 | 0.95 |
| Stroke | -0.05 | 0.11 |  | 0.001 | 0.97 |
| Antihypertensive drugs, no. (%) |  |  |  |  |  |
| Calcium channel blockers | -0.10 | <0.001 |  | -0.08 | <0.001 |
| Angiotensin receptor blockers | 0.02 | 0.52 |  | 0.01 | 0.62 |
| ACE inhibitors | 0.02 | 0.36 |  | -0.33 | 0.16 |
| Diuretics | -0.003 | 0.90 |  | -0.004 | 0.86 |
| Beta-blockers | -0.06 | 0.03 |  | -0.10 | <0.001 |

Abbreviations: R, correlation coefficient; BMI, body mass index; SBP, systolic blood pressure; DBP, diastolic blood pressure; HDL-C, high-density lipoprotein cholesterol; LDL-C, low-density lipoprotein cholesterol; ACE, angiotensin converting enzyme.

*R and *P*-values were calculated by univariate general linear model.

**Supplementary Table S4. Genotype and allele frequencies of 11 variants in all patients.**

| **Variants** | **Genotype** | **Number (Frequency, %)** | **MAF** |
| --- | --- | --- | --- |
|  |  |  |  |
| ***AGT*** | G/G | 1181 (64.6) | A |
| **rs699 G>A** | G/A | 582 (31.8) | 19.5% |
|  | A/A | 66 (3.6) |  |
| ***AGT*** | T/T | 1180 (64.4) | C |
| **rs5051 T>C** | T/C | 584 (31.9) | 19.7% |
|  | C/C | 68 (3.7) |  |
| ***AGTR1*** | A/A | 1646 (89.6) | A |
| **rs5186 A>C** | A/C | 182 (9.9) | 5.4% |
|  | C/C | 9 (0.5) |  |
| ***ADD1*** | G/G | 416 (22.7) | G |
| **rs4961 G>T** | G/T | 899 (49.0) | 47.2% |
|  | T/T | 519 (28.3) |  |
| ***PTPRD*** | C/C | 693 (37.7) | T |
| **rs4742610 C>T** | C/T | 874 (47.6) | 38.5% |
|  | T/T | 270 (14.7) |  |
| ***NR1H3*** | A/A | 1726 (94.0) | G |
| **rs11039149 A>G** | G/A | 111 (6.0) | 3.0% |
|  | G/G | 0 (0.0) |  |
| ***MMP3*** | A/A | 1269 (69.3) | - |
| **rs32025058 A>del** | A/- | 515 (28.1) | 16.7% |
|  | -/- | 48 (2.6) |  |
| ***CACNA1C*** | A/A | 795 (43.6) | G |
| **rs1051375 A>G** | G/A | 809 (44.4) | 34.2% |
|  | G/G | 219 (12.0) |  |
| ***NEDD4L*** | G/G | 1296 (70.7) | A |
| **rs4149601 G>A** | G/A | 496 (27.1) | 15.8% |
|  | A/A | 41 (2.2) |  |
| ***NEDD4L*** | C/C | 1171 (63.8) | G |
| **rs292449 C>G** | C/G | 591 (32.2) | 20.1% |
|  | G/G | 74 (4.0) |  |
| ***ACE2*** | G/G | 733 (39.9) | A |
| **rs2106809 G>A** | G/A | 525 (28.6) | 45.8% |
|  | A/A | 578 (31.5) |  |

Abbreviations: MAF, minor allele frequency.

The full names of the genes are the same as mentioned in the footnote of Supplementary Table S1.

**Supplementary Table S5. Association of genetic variants with the changes in blood pressure in sex stratification.**

| **Variants** | **Changes in BP** | **Allelic association (m vs. M)** | | | |  | **Additive model (mm vs. Mm vs. MM)** | | | |  | **Posterior pairwise comparisons** | | |
| --- | --- | --- | --- | --- | --- | --- | --- | --- | --- | --- | --- | --- | --- | --- |
|  |  |  |  |  |  |  |  |  |  |  |  | **Mm vs. MM** |  | **mm vs. MM** |
|  |  | **Mean (95%CI)*of**  **changes in BP, mm Hg** | | **Mean Difference (95%CI)* of changes in BP, mm Hg** | ***P**** |  | **Mean (95%CI)* of changes in BP, mm Hg** | | | ***P**** |  | ***P**** |  | ***P**** |
| ***AGT* rs699 G>A** | |  |  |  |  |  |  |  |  |  |  |  |  |  |
|  | **Men** | G (81.2%) | A (18.8%) |  |  |  | GG (n=476) | GA (n=222) | AA (n=25) |  |  |  |  |  |
|  | ΔSBP, mm Hg | -2.55 (-4.43, -0.67) | -2.56 (-5.31, 0.18) | -0.01 (-2.62, 2.60) | 0.99 |  | -2.58 (-5.35, 0.19) | -2.33 (-5.66, 1.00) | -3.14 (-11.06, 4.79) | 0.98 |  | 0.88 |  | 0.89 |
|  | ΔDBP, mm Hg | -3.12 (-4.14, -2.09) | -3.58 (-5.07, -2.08) | -0.46 (-1.88, 0.97) | 0.53 |  | -3.04 (-4.55, -1.54) | -3.34 (-5.15, -1.54) | -4.38 (-8.68, -0.08) | 0.80 |  | 0.73 |  | 0.54 |
|  | **Women** | G (80.0%) | A (20.0%) |  |  |  | GG (n=705) | GA (n=360) | AA (n=41) |  |  |  |  |  |
|  | ΔSBP, mm Hg | -2.03 (-3.72, -0.34) | -1.94 (-4.28, 0.41) | 0.09 (-2.00, 2.19) | 0.93 |  | -2.06 (-4.53, 0.41) | -1.84 (-4.75, 1.07) | -2.06 (-8.46, 4.34) | 0.99 |  | 0.87 |  | 1.00 |
|  | ΔDBP, mm Hg | -2.33 (-3.20, -1.45) | -2.73 (-3.94, -1.52) | -0.40 (-1.49, 0.69) | 0.47 |  | -2.20 (-3.47, -0.93) | -2.87 (-4.37, -1.37) | -2.18 (-5.48, 1.12) | 0.60 |  | 0.32 |  | 0.99 |
| ***AGT* rs5051 T>C** | |  |  |  |  |  |  |  |  |  |  |  |  |  |
|  | **Men** | T (80.9%) | C (19.1%) |  |  |  | TT (n=474) | TC (n=224) | CC (n=26) |  |  |  |  |  |
|  | ΔSBP, mm Hg | -2.55 (-4.43, -0.67) | -2.45 (-5.17, 0.28) | 0.11 (-2.49, 2.70) | 0.94 |  | -2.61 (-5.38, 0.17) | -2.24 (-5.54, 1.06) | -2.91 (-10.70, 4.87) | 0.97 |  | 0.82 |  | 0.94 |
|  | ΔDBP, mm Hg | -3.18 (-4.20, -2.16) | -3.55 (-5.04, -2.06) | -0.37 (-1.78, 1.04) | 0.61 |  | -3.10 (-4.60, -1.59) | -3.47 (-5.26, -1.68) | -3.71 (-7.94, 0.51) | 0.89 |  | 0.67 |  | 0.78 |
|  | **Women** | T (80.0%) | C (20.0%) |  |  |  | TT (n=706) | TC (n=360) | CC (n=42) |  |  |  |  |  |
|  | ΔSBP, mm Hg | -2.07 (-3.76, -0.37) | -1.83 (-4.17, 0.51) | 0.23 (-1.86, 2.33) | 0.83 |  | -2.13 (-4.61, 0.35) | -1.73 (-4.63, 1.17) | -1.97 (-8.32, 4.37) | 0.95 |  | 0.76 |  | 0.96 |
|  | ΔDBP, mm Hg | -2.32 (-3.20, -1.44) | -2.67 (-3.88, -1.47) | -0.36 (-1.44, 0.73) | 0.52 |  | -2.20 (-3.48, -0.92) | -2.80 (-4.29, -1.31) | -2.18 (-5.45, 1.09) | 0.67 |  | 0.37 |  | 0.99 |
| ***AGTR1* rs5186 A>C** | |  |  |  |  |  |  |  |  |  |  |  |  |  |
|  | **Men** | A (95.6%) | C (4.4%) |  |  |  | AA (n=666) | AC (n=58) | CC (n=3) |  |  |  |  |  |
|  | ΔSBP, mm Hg | -2.37 (-4.18, -0.56) | -4.46 (-9.51, 0.59) | -2.09 (-7.04, 2.86) | 0.41 |  | -2.27 (-4.84, 0.30) | -5.75 (-11.26, -0.24) | 8.25 (-14.05, 30.55) | 0.28 |  | 0.20 |  | 0.36 |
|  | ΔDBP, mm Hg | -3.16 (-4.15, -2.17) | -4.77 (-7.53, -2.02) | -1.61 (-4.31, 1.09) | 0.24 |  | -3.10 (-4.49, -1.71) | -5.99 (-8.98, -3.01) | 6.95 (-5.14, 19.04) | 0.04 |  | 0.05 |  | 0.10 |
|  | **Women** | A (94.7%) | C (5.3%) |  |  |  | AA (n=980) | AC (n=124) | CC (n=6) |  |  |  |  |  |
|  | ΔSBP, mm Hg | -2.09 (-3.74, -0.44) | -2.04 (-5.69, 1.61) | 0.05 (-3.45, 3.55) | 0.98 |  | -2.07 (-4.43, 0.28) | -2.08 (-6.11, 1.95) | -1.24 (-17.43, 14.96) | 0.99 |  | 1.00 |  | 0.92 |
|  | ΔDBP, mm Hg | -2.43 (-3.28, -1.57) | -2.24 (-4.13, -0.35) | 0.19 (-1.62, 2.00) | 0.84 |  | -2.43 (-3.65, -1.22) | -2.27 (-4.35, -0.19) | -1.93 (-10.27, 6.41) | 0.98 |  | 0.87 |  | 0.91 |
| ***ADD1* rs4961 G>T** | |  |  |  |  |  |  |  |  |  |  |  |  |  |
|  | **Men** | G (46.7%) | T (53.3%) |  |  |  | GG (n=169) | TG (n=341) | TT (n=217) |  |  |  |  |  |
|  | ΔSBP, mm Hg | -1.86 (-4.00, 0.28) | -2.90 (-4.89, -0.91) | -1.05 (-3.08, 0.98) | 0.31 |  | -1.35 (-5.12, 2.42) | -2.20 (-5.12, 0.71) | -3.37 (-6.66, -0.07) | 0.60 |  | 0.65 |  | 0.32 |
|  | ΔDBP, mm Hg | -2.96 (-4.13, -1.79) | -3.44 (-4.52, -2.35) | -0.48 (-1.59, 0.63) | 0.40 |  | -2.54 (-4.59, -0.49) | -3.31 (-4.90 -1.72) | -3.47 (-5.26, -1.68) | 0.67 |  | 0.45 |  | 0.40 |
|  | **Women** | G (47.5%) | T (52.5%) |  |  |  | GG (n=247) | TG (n=558) | TT (n=302) |  |  |  |  |  |
|  | ΔSBP, mm Hg | -2.54 (-4.43, -0.65) | -1.65 (-3.45, 0.14) | 0.89 (-0.80, 2.57) | 0.30 |  | -3.59 (-6.86, -0.32) | -1.62 (-4.21, 0.97) | -1.73 (-4.69, 1.22) | 0.42 |  | 0.20 |  | 0.28 |
|  | ΔDBP, mm Hg | -2.75 (-3.72, -1.77) | -2.14 (-3.06, -1.21) | 0.61 (-0.26, 1.48) | 0.17 |  | -3.27 (-4.96, -1.59) | -2.31 (-3.64, -0.98) | -2.02 (-3.54, -0.50) | 0.34 |  | 0.22 |  | 0.16 |
| ***PTPRD* rs4742610 C>T** | |  |  |  |  |  |  |  |  |  |  |  |  |  |
|  | **Men** | C (61.5%) | T (38.5%) |  |  |  | CC (n=277) | CT (n=340) | TT (n=110) |  |  |  |  |  |
|  | ΔSBP, mm Hg | -2.41 (-4.39, -0.44) | -2.54 (-4.73, -0.35) | -0.12 (-2.21, 1.96) | 0.91 |  | -3.21 (-6.31, -0.12) | -0.55 (-3.59, 2.48) | -4.94 (-9.04, -0.83) | 0.08 |  | 0.10 |  | 0.44 |
|  | ΔDBP, mm Hg | -3.20 (-4.28, -2.12) | -3.29 (-4.48, -2.09) | -0.09 (-1.22, 1.05) | 0.88 |  | -3.55 (-5.24, -1.87) | -2.40 (-4.05, -0.74) | -4.37 (-6.60, -2.13) | 0.18 |  | 0.18 |  | 0.50 |
|  | **Women** | C (61.5%) | T (38.5%) |  |  |  | CC (n=416) | CT (n=534) | TT (n=160) |  |  |  |  |  |
|  | ΔSBP, mm Hg | -2.54 (-4.29, -0.80) | -1.26 (-3.24, 0.72) | 1.29 (-0.44, 3.01) | 0.14 |  | -3.05 (-5.75, -0.35) | -1.62 (-4.28, 1.04) | -0.41 (-4.14, 3.32) | 0.32 |  | 0.28 |  | 0.16 |
|  | ΔDBP, mm Hg | -2.39 (-3.29, -1.48) | -2.47 (-3.50, -1.45) | -0.08 (-0.98, 0.81) | 0.85 |  | -2.40 (-3.79, -1.00) | -2.35 (-3.72, -0.98) | -2.66 (-4.59, -0.74) | 0.94 |  | 0.94 |  | 0.78 |
| ***NR1H3* rs11039149 A>G** | |  |  |  |  |  |  |  |  |  |  |  |  |  |
|  | **Men** | A (97.0%) | G (3.0%) |  |  |  | AA (n=684) | AG (n=43) | GG (n=0) |  |  |  |  |  |
|  | ΔSBP, mm Hg | -2.61 (-4.41, -0.81) | 5.11 (-1.01, 11.24) | 7.72 (1.75, 13.69) | 0.01 |  | -2.74 (-5.27, -0.21) | 5.21 (-1.14, 11.55) | - | 0.01 |  | 0.01 |  | - |
|  | ΔDBP, mm Hg | -3.27 (-4.25, -2.28) | -1.69 (-5.04, 1.65) | 1.57 (-1.69, 4.84) | 0.35 |  | -3.29 (-4.67, -1.91) | -1.68 (-5.14, 1.78) | - | 0.34 |  | 0.34 |  | - |
|  | **Women** | A (96.9%) | G (3.1%) |  |  |  | AA (n=1042) | AG (n=68) | GG (n=0) |  |  |  |  |  |
|  | ΔSBP, mm Hg | -2.23 (-3.87, -0.59) | 2.31 (-2.70, 7.31) | 4.53 (-0.34, 9.41) | 0.07 |  | -2.36 (-4.68, -0.04) | 2.35 (-2.85, 7.54) | - | 0.06 |  | 0.06 |  | - |
|  | ΔDBP, mm Hg | -2.49 (-3.34, -1.64) | -0.08 (-2.67, 2.51) | 2.41 (-0.11, 4.93) | 0.06 |  | -2.56 (-3.76, -1.36) | -0.11 (-2.79, 2.56) | - | 0.06 |  | 0.06 |  | - |

***continued***

| **Variants** | **Changes in BP** | **Allelic association (m vs. M)** | | | |  | **Additive model (mm vs. Mm vs. MM)** | | | |  | **Posterior pairwise comparisons** | | |
| --- | --- | --- | --- | --- | --- | --- | --- | --- | --- | --- | --- | --- | --- | --- |
|  |  |  |  |  |  |  |  |  |  |  |  | **Mm vs. MM** |  | **mm vs. MM** |
|  |  | **Mean (95%CI)*of**  **changes in BP, mm Hg** | | **Mean Difference (95%CI)* of changes in BP, mm Hg** | ***P**** |  | **Mean (95%CI)* of changes in BP, mm Hg** | | | ***P**** |  | ***P**** |  | ***P**** |
| ***MMP3* rs32025058 A>del** | |  |  |  |  |  |  |  |  |  |  |  |  |  |
|  | **Men** | A (83.4%) | - (16.6%) |  |  |  | AA (n=503) | A/- (n=204) | -/- (n=18) |  |  |  |  |  |
|  | ΔSBP, mm Hg | -2.70 (-4.55, -0.85) | -1.62 (-4.52, 1.28) | 1.08 (-1.65, 3.80) | 0.44 |  | -2.78 (-5.46, -0.11) | -3.66 (-7.16, -0.16) | 8.56 (-0.56, 17.68) | 0.04 |  | 0.59 |  | 0.02 |
|  | ΔDBP, mm Hg | -3.41 (-4.42, -2.40) | -2.53 (-4.12, -0.95) | 0.88 (-0.61, 2.37) | 0.25 |  | -3.53 (-4.99, -2.07) | -3.33 (-5.24, -1.42) | 1.52 (-3.46, 6.49) | 0.14 |  | 0.83 |  | 0.05 |
|  | **Women** | A (83.1%) | - (16.9%) |  |  |  | AA (n=766) | A/- (n=311) | -/- (n=30) |  |  |  |  |  |
|  | ΔSBP, mm Hg | -2.14 (-3.82, -0.46) | -2.16 (-4.62, 0.30) | -0.02 (-2.26, 2.22) | 0.99 |  | -2.29 (-4.73, 0.16) | -1.13 (-4.10, 1.85) | -7.03 (-14.43, 0.36) | 0.27 |  | 0.39 |  | 0.21 |
|  | ΔDBP, mm Hg | -2.47 (-3.34, -1.60) | -2.26 (-3.54, -0.99) | 0.21 (-0.96, 1.37) | 0.73 |  | -2.52 (-3.79, -1.26) | -2.13 (-3.67, -0.60) | -2.89 (-6.71, 0.94) | 0.83 |  | 0.58 |  | 0.85 |
| ***CACNA1C* rs1051375 A>G** | | |  |  |  |  |  |  |  |  |  |  |  |  |
|  | **Men** | A (67.1%) | G (32.9%) |  |  |  | AA (n=330) | AG (n=305) | GG (n=84) |  |  |  |  |  |
|  | ΔSBP, mm Hg | -2.47 (-4.44, -0.49) | -2.71 (-5.01, -0.42) | -0.25 (-2.43, 1.94) | 0.82 |  | -2.97 (-6.05, 0.12) | -1.54 (-4.52, 1.45) | -4.93 (-9.64, -0.22) | 0.34 |  | 0.37 |  | 0.42 |
|  | ΔDBP, mm Hg | -3.21 (-4.29, -2.14) | -2.95 (-4.19, -1.71) | 0.26 (-0.92, 1.45) | 0.67 |  | -3.57 (-5.24, -1.90) | -2.53 (-4.15, -0.92) | -3.82 (-6.37, -1.27) | 0.40 |  | 0.22 |  | 0.85 |
|  | **Women** | A (64.9%) | G (35.1%) |  |  |  | AA (n=465) | AG (n=504) | GG (n=135) |  |  |  |  |  |
|  | ΔSBP, mm Hg | -2.57 (-4.32, -0.82) | -1.04 (-3.05, 0.96) | 1.53 (-0.23, 3.29) | 0.09 |  | -3.77 (-6.43, -1.10) | 0.06 (-2.64, 2.75) | -2.62 (-6.49, 1.25) | 0.01 |  | 0.003 |  | 0.56 |
|  | ΔDBP, mm Hg | -2.54 (-3.45, -1.63) | -2.18 (-3.22, -1.15) | 0.36 (-0.56, 1.27) | 0.44 |  | -2.79 (-4.17, -1.42) | -1.96 (-3.35, -0.57) | -2.51 (-4.52, -0.51) | 0.45 |  | 0.21 |  | 0.78 |
| ***NEDD4L* rs4149601 G>A** | |  |  |  |  |  |  |  |  |  |  |  |  |  |
|  | **Men** | G (83.8%) | A (16.2%) |  |  |  | GG (n=507) | GA (n=203) | AA (n=16) |  |  |  |  |  |
|  | ΔSBP, mm Hg | -2.24 (-4.10, -0.38) | -3.43 (-6.34, -0.52) | -1.19 (-3.94, 1.56) | 0.40 |  | -2.06 (-4.79, 0.67) | -3.01 (-6.38, 0.36) | -5.68 (-15.60, 4.24) | 0.67 |  | 0.56 |  | 0.47 |
|  | ΔDBP, mm Hg | -3.14 (-4.15, -2.12) | -3.39 (-4.97, -1.80) | -0.25 (-1.75, 1.25) | 0.74 |  | -3.13 (-4.62, -1.65) | -3.19 (-5.02, -1.36) | -4.38 (-9.77, 1.02) | 0.90 |  | 0.95 |  | 0.65 |
|  | **Women** | G (84.5%) | A (15.5%) |  |  |  | GG (n=789) | GA (n=293) | AA (n=25) |  |  |  |  |  |
|  | ΔSBP, mm Hg | -1.88 (-3.56, -0.20) | -2.97 (-5.52, -0.41) | -1.08 (-3.40, 1.24) | 0.36 |  | -1.63 (-4.06, 0.80) | -3.30 (-6.36, -0.24) | -0.94 (-9.04, 7.16) | 0.46 |  | 0.23 |  | 0.87 |
|  | ΔDBP, mm Hg | -2.42 (-3.28, -1.55) | -2.26 (-3.58, -0.94) | 0.16 (-1.04, 1.35) | 0.80 |  | -2.37 (-3.61, -1.12) | -2.74 (-4.32, -1.17) | 0.38 (-3.78, 4.55) | 0.34 |  | 0.59 |  | 0.19 |
| ***NEDD4L* rs292449 C>G** | |  |  |  |  |  |  |  |  |  |  |  |  |  |
|  | **Men** | C (80.5%) | G (19.5%) |  |  |  | CC (n=471) | CG (n=229) | GG (n=27) |  |  |  |  |  |
|  | ΔSBP, mm Hg | -2.70 (-4.57, -0.83) | -1.55 (-4.27, 1.16) | 1.14 (-1.42, 3.71) | 0.38 |  | -3.11 (-5.86, -0.36) | -0.83 (-4.11, 2.46) | -4.28 (-11.98, 3.43) | 0.31 |  | 0.15 |  | 0.77 |
|  | ΔDBP, mm Hg | -3.18 (-4.20, -2.15) | -3.47 (-4.96, -1.99) | -0.30 (-1.70, 1.10) | 0.68 |  | -3.22 (-4.72, -1.73) | -2.84 (-4.63, -1.05) | -6.01 (-10.20, -1.82) | 0.35 |  | 0.66 |  | 0.19 |
|  | **Women** | C (79.4%) | G (20.6%) |  |  |  | CC (n=700) | CG (n=362) | GG (n=47) |  |  |  |  |  |
|  | ΔSBP, mm Hg | -1.75 (-3.44, -0.06) | -3.37 (-5.71, -1.04) | -1.62 (-3.70, 0.45) | 0.13 |  | -1.37 (-3.84, 1.10) | -3.23 (-6.10, -0.35) | -3.72 (-9.82, 2.39) | 0.31 |  | 0.15 |  | 0.44 |
|  | ΔDBP, mm Hg | -2.31 (-3.19, -1.44) | -2.78 (-3.99, -1.58) | -0.47 (-1.54, 0.60) | 0.39 |  | -2.15 (-3.42, -0.88) | -2.93 (-4.41, -1.45) | -2.21 (-5.36, 0.93) | 0.50 |  | 0.24 |  | 0.97 |
| ***ACE2* rs2106809 G>A** | |  |  |  |  |  |  |  |  |  |  |  |  |  |
|  | **Men** | G (55.4%) | A (44.6%) |  |  |  | - | - | - |  |  |  |  |  |
|  | ΔSBP, mm Hg | -2.60 (-4.59, -0.62) | -2.34 (-4.50, -0.19) | 0.26 (-1.79, 2.31) | 0.80 |  | - | - | - | - |  | - |  | - |
|  | ΔDBP, mm Hg | -3.33 (-4.42, -2.25) | -3.12 (-4.30, -1.95) | 0.21 (-0.90, 1.33) | 0.71 |  | - | - | - | - |  | - |  | - |
|  | **Women** | G (53.5%) | A (46.5%) |  |  |  | GG (n=331) | GA (n=525) | AA (n=254) |  |  |  |  |  |
|  | ΔSBP, mm Hg | -1.52 (-3.32, 0.28) | -2.80 (-4.68, -0.92) | -1.28 (-2.96, 0.40) | 0.14 |  | -1.04 (-3.91, 1.84) | -2.17 (-4.82, 0.49) | -3.41 (-6.57, -0.24) | 0.36 |  | 0.42 |  | 0.15 |
|  | ΔDBP, mm Hg | -2.31 (-3.24, -1.38) | -2.55 (-3.52, -1.57) | -0.23 (-1.10, 0.64) | 0.60 |  | -2.29 (-3.78, -0.81) | -2.30 (-3.67, -0.93) | -2.79 (-4.43, -1.16) | 0.80 |  | 0.99 |  | 0.56 |

Abbreviations: BP, blood pressure; CI, confidence interval; M, major allele; m, minor allele, ΔSBP, changes in systolic blood pressure; ΔDBP, changes in diastolic blood pressure.

*Means, mean differences, 95%CIs and *P*-values were calculated by generalized linear regression model with adjustment for baseline characteristics including age, body mass index, serum creatine, blood pressure, alcohol intake, smoke status and antihypertensive drugs.

The full names of the genes are the same as mentioned in the footnote of Supplementary Table S1.

**Supplementary Table S6. Association of genetic variants with the changes in blood pressure calculated by generalized linear regression model.**

| **Variants** | **Changes in BP** | **Allelic association (m vs. M)** | | | | |  | **Additive model (mm vs. Mm vs. MM)** | | | | |  | **Posterior pairwise comparisons** | | | | |
| --- | --- | --- | --- | --- | --- | --- | --- | --- | --- | --- | --- | --- | --- | --- | --- | --- | --- | --- |
|  |  |  |  |  |  |  |  |  |  |  |  |  |  | **Mm vs. MM** | |  | **mm vs. MM** | |
|  |  | **Mean (95%CI)*of**  **changes in BP, mm Hg** | | **Mean Difference (95%CI)* of changes in BP, mm Hg** | ***P**** | **FDR^†^** |  | **Mean (95%CI)* of changes in BP, mm Hg** | | | ***P**** | **FDR^†^** |  | ***P**** | **FDR^†^** |  | ***P**** | **FDR^†^** |
| ***AGT* rs699 G>A** | | G (94.6%) | A (5.4%) |  |  |  |  | GG (n=1181) | GA (n=582) | AA (n=66) |  |  |  |  |  |  |  |  |
|  | ΔSBP, mm Hg | -2.73 (-4.00, -1.46) | -2.60 (-4.39, -0.80) | 0.14 (-1.50, 1.78) | 0.87 | 0.87 |  | -2.78 (-4.64, -0.93) | -2.40 (-4.60, -0.19) | -3.08 (-8.08, 1.91) | 0.92 | 0.92 |  | 0.70 | 0.96 |  | 0.91 | 1.00 |
|  | ΔDBP, mm Hg | -2.47 (-3.14, -1.80) | -2.88 (-3.82, -1.93) | -0.41 (-1.28, 0.45) | 0.35 | 1.00 |  | -2.36 (-3.34, -1.39) | -2.88 (-4.03, -1.72) | -2.85 (-5.47, -0.22) | 0.61 | 1.00 |  | 0.34 | 0.94 |  | 0.72 | 1.00 |
| ***AGT* rs5051 T>C** | | T (80.3%) | C (19.7%) |  |  |  |  | TT (n=1180) | TC (n=584) | CC (n=68) |  |  |  |  |  |  |  |  |
|  | ΔSBP, mm Hg | -2.76 (-4.03, -1.49) | -2.49 (-4.27, -0.70) | 0.28 (-1.36, 1.91) | 0.74 | 1.00 |  | -2.86 (-4.72, -0.99) | -2.29 (-4.48, -0.10) | -2.98 (-7.92, 1.95) | 0.85 | 1.00 |  | 0.58 | 0.91 |  | 0.96 | 1.00 |
|  | ΔDBP, mm Hg | -2.49 (-3.16, -1.82) | -2.84 (-3.78, -1.90) | -0.35 (-1.21, 0.51) | 0.42 | 0.77 |  | -2.39 (-3.36, -1.41) | -2.88 (-4.03, -1.73) | -2.63 (-5.22, -0.04) | 0.65 | 1.00 |  | 0.36 | 0.66 |  | 0.85 | 1.00 |
| ***AGTR1* rs5186 A>C** | | A (80.5%) | C (19.5%) |  |  |  |  | AA (n=1646) | AC (n=182) | CC (n=9) |  |  |  |  |  |  |  |  |
|  | ΔSBP, mm Hg | -2.69 (-3.92, -1.46) | -3.14 (-6.10, -0.18) | -0.45 (-3.31, 2.41) | 0.76 | 0.93 |  | -2.63 (-4.38, -0.87) | -3.58 (-6.83, -0.32) | 1.76 (-11.36, 14.88) | 0.66 | 1.00 |  | 0.54 | 0.99 |  | 0.51 | 1.00 |
|  | ΔDBP, mm Hg | -2.54 (-3.19, -1.89) | -2.98 (-4.54, -1.41) | -0.44 (-1.94, 1.07) | 0.57 | 0.90 |  | -2.49 (-3.41, -1.58) | -3.40 (-5.11, -1.69) | 1.40 (-5.49, 8.29) | 0.29 | 1.00 |  | 0.27 | 0.99 |  | 0.27 | 1.00 |
| ***ADD1* rs4961 G>T** | | G (47.2%) | T (52.8%) |  |  |  |  | GG (n=416) | TG (n=899) | TT (n=519) |  |  |  |  |  |  |  |  |
|  | ΔSBP, mm Hg | -2.76 (-4.19, -1.34) | -2.62 (-3.96, -1.27) | 0.15 (-1.15, 1.45) | 0.82 | 0.90 |  | -3.19 (-5.67, -0.71) | -2.32 (-4.28, -0.37) | -2.87 (-5.09, -0.66) | 0.74 | 1.00 |  | 0.47 | 1.00 |  | 0.81 | 1.00 |
|  | ΔDBP, mm Hg | -2.67 (-3.42, -1.92) | -2.47 (-3.18, -1.76) | 0.20 (-0.48, 0.89) | 0.57 | 0.78 |  | -2.83 (-4.13, -1.52) | -2.52 (-3.55, -1.50) | -2.42 (-3.59, -1.26) | 0.84 | 0.92 |  | 0.63 | 0.69 |  | 0.56 | 1.00 |
| ***PTPRD* rs4742610 C>T** | | C (61.5%) | T (38.5%) |  |  |  |  | CC (n=693) | CT (n=874) | TT (n=270) |  |  |  |  |  |  |  |  |
|  | ΔSBP, mm Hg | -3.01 (-4.33, -1.69) | -2.22 (-3.69, -0.74) | 0.79 (-0.54, 2.13) | 0.24 | 0.66 |  | -3.71 (-5.76, -1.66) | -1.69 (-3.70, 0.33) | -2.76 (-5.54, 0.02) | 0.14 | 0.51 |  | 0.05 | 0.18 |  | 0.51 | 1.00 |
|  | ΔDBP, mm Hg | -2.55 (-3.25, -1.85) | -2.59 (-3.37, -1.81) | -0.04 (-0.74, 0.66) | 0.91 | 1.00 |  | -2.72 (-3.79, -1.64) | -2.21 (-3.27, -1.15) | -3.10 (-4.56, -1.64) | 0.40 | 1.00 |  | 0.35 | 0.77 |  | 0.61 | 1.00 |
| ***NR1H3* rs11039149 A>G** | | A (97.0%) | G (3.0%) |  |  |  |  | AA (n=1726) | AG (n=111) | GG (n=0) |  |  |  |  |  |  |  |  |
|  | ΔSBP, mm Hg | -2.86 (-4.08, -1.63) | 2.91 (-0.98, 6.80) | 5.77 (1.98, 9.56) | 0.003 | 0.03 |  | -2.99 (-4.71, -1.26) | 2.95 (-1.08, 6.98) | - | 0.002 | 0.02 |  | 0.002 | 0.02 |  | - | 1.00 |
|  | ΔDBP, mm Hg | -2.62 (-3.26, -1.97) | -0.54 (-2.59, 1.52) | 2.08 (0.08, 4.08) | 0.04 | 0.44 |  | -2.66 (-3.57, -1.75) | -0.56 (-2.68, 1.57) | - | 0.04 | 0.44 |  | 0.04 | 0.44 |  | - | 1.00 |
| ***MMP3* rs32025058 A>del** | | A (83.3%) | - (16.7%) |  |  |  |  | AA (n=1269) | A/- (n=515) | -/- (n=48) |  |  |  |  |  |  |  |  |
|  | ΔSBP, mm Hg | -2.83 (-4.08, -1.57) | -2.50 (-4.38, -0.61) | 0.33 (-1.41, 2.07) | 0.71 | 1.00 |  | -2.86 (-4.69, -1.04) | -2.64 (-4.92, -0.35) | -1.48 (-7.24, 4.29) | 0.88 | 0.97 |  | 0.83 | 0.83 |  | 0.64 | 1.00 |
|  | ΔDBP, mm Hg | -2.66 (-3.33, -2.00) | -2.22 (-3.22, -1.23) | 0.44 (-0.48, 1.36) | 0.35 | 1.00 |  | -2.72 (-3.68, -1.76) | -2.43 (-3.63, -1.23) | -1.15 (-4.18, 1.89) | 0.54 | 1.00 |  | 0.60 | 0.83 |  | 0.31 | 1.00 |
| ***CACNA1C* rs1051375 A>G** | | A (65.8%) | G (34.2%) |  |  |  |  | AA (n=795) | AG (n=809) | GG (n=219) |  |  |  |  |  |  |  |  |
|  | ΔSBP, mm Hg | -3.03 (-4.34, -1.71) | -2.18 (-3.70, -0.66) | 0.85 (-0.53, 2.22) | 0.23 | 0.84 |  | -3.93 (-5.95, -1.91) | -1.09 (-3.11, 0.93) | -4.00 (-7.01, -1.00) | 0.01 | 0.06 |  | 0.004 | 0.02 |  | 0.96 | 0.96 |
|  | ΔDBP, mm Hg | -2.61 (-3.31, -1.92) | -2.29 (-3.09, -1.48) | 0.33 (-0.40, 1.05) | 0.38 | 1.00 |  | -2.91 (-3.97, -1.85) | -1.97 (-3.03, -0.91) | -2.84 (-4.42, -1.26) | 0.17 | 0.94 |  | 0.07 | 0.39 |  | 0.94 | 0.94 |
| ***NEDD4L* rs4149601 G>A** | | G (84.2%) | A (15.8%) |  |  |  |  | GG (n=1296) | GA (n=496) | AA (n=41) |  |  |  |  |  |  |  |  |
|  | ΔSBP, mm Hg | -2.49 (-3.75, -1.23) | -3.66 (-5.59, -1.73) | -1.17 (-2.96, 0.61) | 0.20 | 1.00 |  | -2.24 (-4.07, -0.41) | -3.62 (-5.90, -1.34) | -3.53 (-9.83, 2.76) | 0.41 | 1.00 |  | 0.19 | 0.52 |  | 0.68 | 1.00 |
|  | ΔDBP, mm Hg | -2.52 (-3.18, -1.85) | -2.54 (-3.55, -1.52) | -0.02 (-0.96, 0.92) | 0.96 | 0.96 |  | -2.46 (-3.42, -1.50) | -2.74 (-3.93, -1.54) | -1.23 (-4.53, 2.08) | 0.65 | 0.89 |  | 0.62 | 0.76 |  | 0.46 | 1.00 |
| ***NEDD4L* rs292449 C>G** | | C (79.9%) | G (20.1%) |  |  |  |  | CC (n=1171) | CG (n=591) | GG (n=74) |  |  |  |  |  |  |  |  |
|  | ΔSBP, mm Hg | -2.57 (-3.83, -1.30) | -3.24 (-5.02, -1.46) | -0.68 (-2.29, 0.94) | 0.41 | 0.75 |  | -2.50 (-4.35, -0.64) | -2.76 (-4.94, -0.59) | -4.85 (-9.64, -0.07) | 0.61 | 1.00 |  | 0.79 | 0.97 |  | 0.33 | 1.00 |
|  | ΔDBP, mm Hg | -2.48 (-3.15, -1.82) | -2.86 (-3.80, -1.92) | -0.38 (-1.23, 0.47) | 0.38 | 0.84 |  | -2.42 (-3.39, -1.44) | -2.72 (-3.86, -1.57) | -3.39 (-5.90, -0.87) | 0.67 | 0.82 |  | 0.57 | 0.90 |  | 0.44 | 1.00 |
| ***ACE2* rs2106809 G>A** | | G (54.2%) | A (45.8%) |  |  |  |  | GG (n=733) | GA (n=525) | AA (n=578) |  |  |  |  |  |  |  |  |
|  | ΔSBP, mm Hg | -2.40 (-3.74, -1.05) | -3.16 (-4.58, -1.73) | -0.76 (-2.06, 0.55) | 0.26 | 0.57 |  | -2.30 (-4.32, -0.28) | -2.60 (-5.08, -0.13) | -3.35 (-5.56, -1.15) | 0.63 | 1.00 |  | 0.81 | 0.89 |  | 0.34 | 1.00 |
|  | ΔDBP, mm Hg | -2.55 (-3.26, -1.84) | -2.59 (-3.34, -1.83) | -0.03 (-0.72, 0.65) | 0.92 | 1.00 |  | -2.60 (-3.66, -1.54) | -2.32 (-3.62, -1.02) | -2.68 (-3.84, -1.52) | 0.87 | 0.87 |  | 0.68 | 0.68 |  | 0.88 | 0.98 |

Abbreviations: BP, blood pressure; CI, confidence interval; M, major allele; m, minor allele; FDR, false discovery rate, ΔSBP, changes in systolic blood pressure; ΔDBP, changes in diastolic blood pressure.

*Means, mean differences, 95%CIs and *P*-values were calculated by generalized linear regression model with adjustment for baseline characteristics including age, sex, body mass index, serum creatine, blood pressure, alcohol intake, smoke status and antihypertensive drugs.

†FDRs were calculated by the Benjamin & Hochberg method.

The full names of the genes are the same as mentioned in the footnote of Supplementary Table S1.

**Supplementary Table S7. Association of genetic variants with the changes in blood pressure calculated by multivariable linear regression model.**

| **Variants** | **Changes in BP** | **Allelic association (m vs. M)** | | | |  | **Additive Model (mm vs. Mm vs. MM)** | | | |  | **Posterior pairwise comparisons (Mm vs. MM)** | | | |  | **Posterior pairwise comparisons (mm vs. MM)** | | | |
| --- | --- | --- | --- | --- | --- | --- | --- | --- | --- | --- | --- | --- | --- | --- | --- | --- | --- | --- | --- | --- |
|  |  | **Beta*** | **SE*** | ***P**** | **FDR^†^** |  | **Beta*** | **SE*** | ***P**** | **FDR^†^** |  | **Beta*** | **SE*** | ***P**** | **FDR^†^** |  | **Beta*** | **SE*** | ***P**** | **FDR^†^** |
| ***AGT* rs699 G>A** | |  |  |  |  |  |  |  |  |  |  |  |  |  |  |  |  |  |  |  |
| ΔSBP, mm Hg | | 0.14 | 0.84 | 0.87 | 0.87 |  | 0.18 | 0.60 | 0.77 | 0.84 |  | 0.37 | 1.02 | 0.72 | 0.88 |  | -0.25 | 1.28 | 0.84 | 1.00 |
| ΔDBP, mm Hg | | -0.41 | 0.44 | 0.35 | 1.00 |  | -0.41 | 0.32 | 0.19 | 0.70 |  | -0.52 | 0.54 | 0.33 | 0.73 |  | -0.20 | 0.69 | 0.77 | 1.00 |
| ***AGT* rs5051 T>C** | |  |  |  |  |  |  |  |  |  |  |  |  |  |  |  |  |  |  |  |
| ΔSBP, mm Hg | | 0.28 | 0.84 | 0.74 | 1.00 |  | 0.32 | 0.60 | 0.59 | 0.81 |  | 0.56 | 1.02 | 0.59 | 0.92 |  | -0.16 | 1.26 | 0.90 | 1.00 |
| ΔDBP, mm Hg | | -0.35 | 0.44 | 0.42 | 0.78 |  | -0.35 | 0.31 | 0.27 | 0.49 |  | -0.50 | 0.54 | 0.35 | 0.65 |  | -0.08 | 0.68 | 0.91 | 1.00 |
| ***AGTR1* rs5186 A>C** | |  |  |  |  |  |  |  |  |  |  |  |  |  |  |  |  |  |  |  |
| ΔSBP, mm Hg | | -0.45 | 1.46 | 0.76 | 0.93 |  | -0.41 | 1.02 | 0.69 | 0.84 |  | -0.95 | 1.57 | 0.55 | 1.00 |  | 2.13 | 3.37 | 0.53 | 1.00 |
| ΔDBP, mm Hg | | -0.44 | 0.77 | 0.57 | 0.79 |  | -0.42 | 0.54 | 0.44 | 0.60 |  | -0.91 | 0.83 | 0.27 | 0.99 |  | 1.96 | 1.78 | 0.27 | 1.00 |
| ***ADD1* rs4961 G>T** | |  |  |  |  |  |  |  |  |  |  |  |  |  |  |  |  |  |  |  |
| ΔSBP, mm Hg | | 0.15 | 0.66 | 0.83 | 0.91 |  | -0.12 | 0.47 | 0.79 | 0.79 |  | 0.96 | 1.20 | 0.42 | 0.93 |  | 0.24 | 0.67 | 0.72 | 1.00 |
| ΔDBP, mm Hg | | 0.20 | 0.35 | 0.57 | 0.89 |  | -0.20 | 0.25 | 0.42 | 0.66 |  | 0.36 | 0.63 | 0.57 | 0.89 |  | 0.21 | 0.34 | 0.54 | 1.00 |
| ***PTPRD* rs4742610 C>T** | |  |  |  |  |  |  |  |  |  |  |  |  |  |  |  |  |  |  |  |
| ΔSBP, mm Hg | | 0.79 | 0.68 | 0.24 | 0.67 |  | 0.83 | 0.48 | 0.09 | 0.23 |  | 2.13 | 1.03 | 0.04 | 0.15 |  | 0.38 | 0.72 | 0.59 | 1.00 |
| ΔDBP, mm Hg | | -0.04 | 0.36 | 0.91 | 1.00 |  | -0.04 | 0.26 | 0.89 | 1.00 |  | 0.56 | 0.54 | 0.30 | 0.82 |  | -0.21 | 0.38 | 0.58 | 0.97 |
| ***NR1H3* rs11039149 A>G** | |  |  |  |  |  |  |  |  |  |  |  |  |  |  |  |  |  |  |  |
| ΔSBP, mm Hg | | 5.77 | 1.94 | 0.003 | 0.03 |  | 5.97 | 1.39 | <0.001 | <0.001 |  | 5.97 | 1.39 | <0.001 | <0.001 |  | - | - | - | - |
| ΔDBP, mm Hg | | 2.08 | 1.02 | 0.04 | 0.46 |  | 2.14 | 0.74 | 0.04 | 0.44 |  | 2.14 | 0.74 | 0.04 | 0.44 |  | - | - | - | - |
| ***MMP3* rs32025058 A>del** | |  |  |  |  |  |  |  |  |  |  |  |  |  |  |  |  |  |  |  |
| ΔSBP, mm Hg | | 0.33 | 0.89 | 0.71 | 1.00 |  | 0.37 | 0.63 | 0.56 | 0.87 |  | 0.23 | 1.05 | 0.83 | 0.83 |  | 0.70 | 1.48 | 0.64 | 1.00 |
| ΔDBP, mm Hg | | 0.44 | 0.47 | 0.35 | 1.00 |  | 0.45 | 0.33 | 0.18 | 0.97 |  | 0.27 | 0.55 | 0.62 | 0.76 |  | 0.72 | 0.78 | 0.36 | 1.00 |
| ***CACNA1C* rs1051375 A>G** | |  |  |  |  |  |  |  |  |  |  |  |  |  |  |  |  |  |  |  |
| ΔSBP, mm Hg | | 0.85 | 0.70 | 0.23 | 0.83 |  | 0.86 | 0.49 | 0.08 | 0.30 |  | 2.85 | 1.00 | 0.00 | 0.02 |  | -0.04 | 0.75 | 0.95 | 0.95 |
| ΔDBP, mm Hg | | 0.33 | 0.37 | 0.38 | 1.00 |  | 0.32 | 0.26 | 0.22 | 0.61 |  | 0.95 | 0.52 | 0.07 | 0.38 |  | 0.00 | 0.40 | 1.00 |  |
| ***NEDD4L* rs4149601 G>A** | |  |  |  |  |  |  |  |  |  |  |  |  |  |  |  |  |  |  |  |
| ΔSBP, mm Hg | | -1.17 | 0.91 | 0.20 | 1.00 |  | -1.17 | 0.65 | 0.07 | 0.40 |  | -1.38 | 1.07 | 0.20 | 0.54 |  | -0.69 | 1.60 | 0.67 | 1.00 |
| ΔDBP, mm Hg | | -0.02 | 0.48 | 0.96 | 0.96 |  | -0.01 | 0.34 | 0.97 | 0.97 |  | -0.27 | 0.56 | 0.63 | 0.69 |  | 0.61 | 0.84 | 0.47 | 1.00 |
| ***NEDD4L* rs292449 C>G** | |  |  |  |  |  |  |  |  |  |  |  |  |  |  |  |  |  |  |  |
| ΔSBP, mm Hg | | -0.68 | 0.83 | 0.41 | 0.76 |  | -0.64 | 0.59 | 0.27 | 0.50 |  | -0.27 | 1.02 | 0.79 | 0.87 |  | -1.23 | 1.19 | 0.30 | 1.00 |
| ΔDBP, mm Hg | | -0.38 | 0.44 | 0.38 | 0.85 |  | -0.37 | 0.31 | 0.23 | 0.50 |  | -0.30 | 0.54 | 0.57 | 0.79 |  | -0.49 | 0.63 | 0.44 | 1.00 |
| ***ACE2* rs2106809 G>A** | |  |  |  |  |  |  |  |  |  |  |  |  |  |  |  |  |  |  |  |
| ΔSBP, mm Hg | | -0.76 | 0.67 | 0.26 | 0.56 |  | -0.53 | 0.39 | 0.18 | 0.40 |  | -0.73 | 1.37 | 0.60 | 0.82 |  | -0.54 | 0.57 | 0.34 | 1.00 |
| ΔDBP, mm Hg | | -0.03 | 0.35 | 0.92 | 1.00 |  | -0.03 | 0.21 | 0.90 | 0.99 |  | 0.18 | 0.72 | 0.80 | 0.80 |  | -0.05 | 0.30 | 0.87 | 1.00 |

Abbreviations: BP, blood pressure; Beta, regression beta coefficients; SE, standard error, ΔSBP, changes in systolic blood pressure; ΔDBP, changes in diastolic blood pressure.

*Betas, SEs and *P*-values were calculated by multiple linear regression model with adjustment for baseline characteristics including age, sex, body mass index, serum creatine, blood pressure, alcohol intake, smoke status and antihypertensive drugs.

†FDRs were calculated by the Benjamin & Hochberg method.

The full names of the genes are the same as mentioned in the footnote of Supplementary Table S1.

**Supplementary Table S8. Association of two-variants interactions between *NR1H3* variant rs11039149 A>G and other variants with the changes in blood pressure.**

| **Variant 1** | **Variant 2** | **ΔSBP, mm Hg** | |  | **ΔDBP, mm Hg** | |
| --- | --- | --- | --- | --- | --- | --- |
|  |  | ***P* for interaction*** | **FDR^†^** |  | ***P* for interaction*** | **FDR^†^** |
| rs11039149 A>G | rs699 G>A | 0.41 | 1.00 |  | 0.37 | 0.54 |
| rs11039149 A>G | rs5051 T>C | 0.35 | 1.00 |  | 0.52 | 0.64 |
| rs11039149 A>G | rs5186 A>C | 0.83 | 0.92 |  | 0.13 | 0.64 |
| rs11039149 A>G | rs4961 G>T | 0.76 | 0.95 |  | 0.69 | 0.76 |
| rs11039149 A>G | rs4742610 C>T | 0.83 | 0.83 |  | 0.15 | 0.38 |
| rs11039149 A>G | rs32025058 A>del | 0.52 | 0.87 |  | 0.17 | 0.34 |
| rs11039149 A>G | rs1051375 A>G | 0.44 | 0.88 |  | 0.92 | 0.92 |
| rs11039149 A>G | rs4149601 G>A | 0.57 | 0.81 |  | 0.12 | 1.00 |
| rs11039149 A>G | rs292449 C>G | 0.35 | 1.00 |  | 0.13 | 0.45 |
| rs11039149 A>G | rs2106809 G>A | 0.33 | 1.00 |  | 0.31 | 0.52 |

Abbreviations: *NR1H3*, nuclear receptor subfamily 1 group H member 3; FDR, false discovery rate; ΔSBP, changes in systolic blood pressure; ΔDBP, changes in diastolic blood pressure.

The epistasis analysis was performed by using the PLINK 1.9 under the 2×3 joint genotype count tables (Variant 1: rs11039149 AA & AG × Variant 2: MM & Mm & mm) to calculate the *P*-values for interaction for the association between two-variants interaction and the changes in blood pressure.

* *P*-values for interaction were calculated by multiple linear regression model with adjustment for baseline characteristics including age, sex, body mass index, serum creatine, blood pressure, alcohol intake, smoke status and antihypertensive drugs.

†FDRs were calculated by the Benjamin & Hochberg method.

**Supplementary Table S9. Association of *NR1H3* variant rs11039149 A>G with the changes in blood pressure calculated by multiple linear mixing model.**

| **Changes in BP** | **Genotype** | **Mean (95%CI)* of**  **changes in BP, mm Hg** | **Mean Difference (95%CI)* of changes in BP, mm Hg** | ***P**** |
| --- | --- | --- | --- | --- |
| ***NR1H3* rs11039149 A>G** | |  |  |  |
| **ΔSBP, mm Hg** | AA (n=1726) | -2.84 (-4.61, -1.07) | 5.00 (1.09, 8.91) | 0.01 |
|  | AG (n=111) | 2.16 (-1.94, 6.26) |  |  |
| **ΔDBP, mm Hg** | AA (n=1726) | -2.71 (-3.63, -1.79) | 2.10 (0.05, 4.15) | 0.05 |
|  | AG (n=111) | -0.61 (-2.76, 1.54) |  |  |

Abbreviations: *NR1H3*, nuclear receptor subfamily 1 group H member 3; BP, blood pressure; CI, confidence interval; ΔSBP, changes in systolic blood pressure; ΔDBP, changes in diastolic blood pressure.

*Means, mean differences, 95%CIs and *P*-values were calculated by multiple linear mixing model. Baseline characteristics of age, sex, body mass index, serum creatine, blood pressure, alcohol intake, smoke status and antihypertensive drugs were adjusted as fixed effect, entry time and center were adjusted as random effect. Mean differences were calculated between AG and AA genotypes.

**Supplementary Table S10. Baseline characteristics of patients of *NR1H3* variant rs11039149 AA and AG genotypes.**

| **Characteristics** | **AA** | **AG** | ***P**** |
| --- | --- | --- | --- |
|  | **(n=1726)** | **(n=111)** |  |
| Age, years | 63.0 ± 9.6 | 63.2 ± 10.4 | 0.86 |
| Men, no. (%) | 684 (39.6) | 43 (38.7) | 0.85 |
| BMI, kg/m^2^ | 26.4 ± 3.4 | 25.7 ± 3.2 | 0.03 |
| SBP, mm Hg |  |  |  |
| Baseline | 156 ± 22 | 156 ± 22 | 0.86 |
| Follow-up | 151 ± 22 | 157 ± 23 | 0.004 |
| DBP, mm Hg |  |  |  |
| Baseline | 89.5 ± 12.2 | 90.0 ± 12.1 | 0.70 |
| Follow-up | 85.9 ± 11.9 | 88.0 ± 12.5 | 0.07 |
| Lipids, mmol/L | | | |
| Total cholesterol | 5.54 ± 1.05 | 5.43 ± 0.95 | 0.24 |
| Triglycerides | 1.62 (1.12-2.34) | 1.47 (1.02-2.15) | 0.19 |
| HDL-C | 1.36 ± 0.31 | 1.40 ± 0.33 | 0.26 |
| LDL-C | 3.56 ± 0.83 | 3.42 ± 0.76 | 0.10 |
| Fasting serum glucose, mmol/L | 5.69 (5.24-6.47) | 5.58 (5.13-6.37) | 0.15 |
| Serum creatinine, μmol/L | 76.2 ± 19.9 | 81.5 ± 26.8 | 0.01 |
| Cigarette smoking, no. (%) | 548 (31.7) | 41 (36.9) | 0.26 |
| Alcohol intake, no. (%) | 543 (31.5) | 38 (34.2) | 0.54 |
| Medical history, no. (%) | | | |
| Coronary heart disease | 478 (27.7) | 35 (31.5) | 0.38 |
| Diabetes | 367 (21.3) | 23 (20.7) | 0.89 |
| Stroke | 325 (18.8) | 24 (21.6) | 0.47 |
| Antihypertensive drugs, no. (%) |  |  |  |
| Calcium channel blockers | 1112 (64.4) | 72 (64.9) | 0.93 |
| Angiotensin receptor blockers | 922 (53.4) | 55 (49.5) | 0.43 |
| ACE inhibitors | 209 (12.1) | 12 (10.8) | 0.68 |
| Diuretics | 390 (22.6) | 24 (21.6) | 0.81 |
| Beta-blockers | 360 (20.9) | 20 (18.0) | 0.47 |

Abbreviations: *NR1H3*, nuclear receptor subfamily 1 group H member 3; BMI, body mass index; SBP, systolic blood pressure; DBP, diastolic blood pressure; HDL-C, high-density lipoprotein cholesterol; LDL-C, low-density lipoprotein cholesterol; ACE, angiotensin converting enzyme.

Values were presented as mean ± standard deviation, number (percentage), or median (interquartile range). The t-test was used for comparison of continuous variables, the chi-square test for categorical variables, and the Mann-Whitney U test for triglycerides and fasting serum glucose.

**P*-values were calculated between the AA and AG genotypes.

**Supplementary Table S11. Association of genetic variants with the blood pressure response to antihypertensive drugs therapy.**

| **Variants** | **Changes in BP** | **Mean (95%CI)* of changes in BP, mm Hg** | | **Mean Difference (95%CI)* of changes in BP, mm Hg** | ***P**** | **FDR^†^** |
| --- | --- | --- | --- | --- | --- | --- |
| ***AGT* rs699 G>A** | |  |  |  |  |  |
| CCBs use (n=1182) | | GG (n=748) | GA+AA (n=434) |  |  |  |
|  | ΔSBP, mm Hg | -3.65 (-6.20, -1.10) | -3.17 (-5.98, -0.36) | 0.48 (-2.04, 3.01) | 0.71 | 0.97 |
|  | ΔDBP, mm Hg | -2.80 (-4.12, -1.47) | -3.69 (-5.14, -2.23) | -0.89 (-2.20, 0.42) | 0.18 | 1.00 |
| ARBs or ACEIs use (n=1163) | | GG (n=769) | GA+AA (n=394) |  |  |  |
|  | ΔSBP, mm Hg | -4.13 (-6.06, -2.21) | -3.69 (-6.08, -1.31) | 0.44 (-2.05, 2.93) | 0.73 | 0.80 |
|  | ΔDBP, mm Hg | -2.86 (-3.86, -1.85) | -3.35 (-4.60, -2.10) | -0.49 (-1.80, 0.81) | 0.46 | 0.84 |
| Diuretics use (n=413) | | GG (n=263) | GA+AA (n=150) |  |  |  |
|  | ΔSBP, mm Hg | -2.90 (-6.85, 1.06) | -2.61 (-7.16, 1.94) | 0.28 (-4.00, 4.56) | 0.90 | 0.99 |
|  | ΔDBP, mm Hg | -0.84 (-2.81, 1.13) | -1.88 (-4.14, 0.39) | -1.04 (-3.17, 1.09) | 0.34 | 0.47 |
| Beta-blockers use (n=379) | | GG (n=242) | GA+AA (n=137) |  |  |  |
|  | ΔSBP, mm Hg | -5.18 (-9.32, -1.04) | -2.78 (-7.74, 2.17) | 2.40 (-2.35, 7.15) | 0.32 | 0.71 |
|  | ΔDBP, mm Hg | -4.38 (-6.55, -2.21) | -4.37 (-6.96, -1.77) | 0.01 (-2.48, 2.50) | 0.99 | 0.99 |
| ***AGT* rs5051 T>C** | |  |  |  |  |  |
| CCBs use (n=1183) | | TT (n=748) | TC+CC (n=435) |  |  |  |
|  | ΔSBP, mm Hg | -3.71 (-6.26, -1.15) | -3.10 (-5.91, -0.29) | 0.61 (-1.92, 3.13) | 0.64 | 1.00 |
|  | ΔDBP, mm Hg | -2.79 (-4.11, -1.47) | -3.67 (-5.13, -2.22) | -0.88 (-2.19, 0.42) | 0.19 | 0.68 |
| ARBs or ACEIs use (n=1165) | | TT (n=767) | TC+CC (n=398) |  |  |  |
|  | ΔSBP, mm Hg | -4.13 (-6.06, -2.20) | -3.60 (-5.97, -1.22) | 0.53 (-1.95, 3.02) | 0.68 | 0.93 |
|  | ΔDBP, mm Hg | -2.86 (-3.87, -1.85) | -3.32 (-4.56, -2.08) | -0.46 (-1.76, 0.84) | 0.49 | 0.77 |
| Diuretics use (n=413) | | TT (n=261) | TC+CC (n=152) |  |  |  |
|  | ΔSBP, mm Hg | -2.87 (-6.85, 1.10) | -2.70 (-7.23, 1.83) | 0.17 (-4.10, 4.45) | 0.94 | 0.94 |
|  | ΔDBP, mm Hg | -0.86 (-2.83, 1.12) | -1.81 (-4.06, 0.44) | -0.95 (-3.08, 1.17) | 0.38 | 0.46 |
| Beta-blockers use (n=379) | | TT (n=241) | TC+CC (n=138) |  |  |  |
|  | ΔSBP, mm Hg | -5.33 (-9.49, -1.18) | -2.56 (-7.48, 2.35) | 2.77 (-1.96, 7.50) | 0.25 | 0.69 |
|  | ΔDBP, mm Hg | -4.40 (-6.58, -2.22) | -4.33 (-6.90, -1.75) | 0.08 (-2.40, 2.55) | 0.95 | 1.00 |
| ***AGTR1* rs5186 A>C** | |  |  |  |  |  |
| CCBs use (n=1184) | | AA (n=1066) | AC+CC (n=118) |  |  |  |
|  | ΔSBP, mm Hg | -3.37 (-5.75, -0.98) | -3.89 (-8.25, 0.47) | -0.52 (-4.59, 3.55) | 0.80 | 0.98 |
|  | ΔDBP, mm Hg | -3.02 (-4.25, -1.78) | -4.03 (-6.28, -1.77) | -1.01 (-3.11, 1.10) | 0.35 | 0.77 |
| ARBs or ACEIs use (n=1170) | | AA (n=1051) | AC+CC (n=119) |  |  |  |
|  | ΔSBP, mm Hg | -3.80 (-5.57, -2.04) | -4.76 (-8.68, -0.85) | -0.96 (-4.85, 2.93) | 0.63 | 0.99 |
|  | ΔDBP, mm Hg | -2.92 (-3.84, -1.99) | -3.95 (-6.00, -1.90) | -1.03 (-3.07, 1.01) | 0.32 | 0.71 |
| Diuretics use (n=414) | | AA (n=372) | AC+CC (n=42) |  |  |  |
|  | ΔSBP, mm Hg | -2.73 (-6.49, 1.03) | -3.59 (-10.49, 3.30) | -0.86 (-7.67, 5.95) | 0.80 | 0.98 |
|  | ΔDBP, mm Hg | -0.98 (-2.85, 0.89) | -2.78 (-6.21, 0.65) | -1.80 (-5.18, 1.58) | 0.30 | 0.54 |
| Beta-blockers use (n=380) | | AA (n=342) | AC+CC (n=38) |  |  |  |
|  | ΔSBP, mm Hg | -4.81 (-8.69, -0.93) | 0.53 (-7.25, 8.32) | 5.34 (-2.16, 12.84) | 0.16 | 0.60 |
|  | ΔDBP, mm Hg | -4.50 (-6.53, -2.47) | -2.90 (-6.97, 1.18) | 1.60 (-2.32, 5.53) | 0.42 | 1.00 |
| ***ADD1* rs4961 G>T** | |  |  |  |  |  |
| CCBs use (n=1182) | | GG (n=272) | GT+TT (n=910) |  |  |  |
|  | ΔSBP, mm Hg | -3.56 (-6.89, -0.23) | -3.36 (-5.78, -0.95) | 0.20 (-2.70, 3.09) | 0.90 | 0.90 |
|  | ΔDBP, mm Hg | -3.43 (-5.16, -1.71) | -3.04 (-4.29, -1.79) | 0.39 (-1.11, 1.89) | 0.61 | 0.95 |
| ARBs or ACEIs use (n=1168) | | GG (n=264) | GT+TT (n=904) |  |  |  |
|  | ΔSBP, mm Hg | -6.37 (-9.19, -3.54) | -3.24 (-5.05, -1.43) | 3.13 (0.32, 5.93) | 0.03 | 0.32 |
|  | ΔDBP, mm Hg | -3.99 (-5.47, -2.51) | -2.75 (-3.70, -1.80) | 1.24 (-0.23, 2.71) | 0.10 | 0.36 |
| Diuretics use (n=414) | | GG (n=92) | GT+TT (n=322) |  |  |  |
|  | ΔSBP, mm Hg | -4.56 (-10.13, 1.02) | -2.56 (-6.27, 1.15) | 2.00 (-2.96, 6.96) | 0.43 | 0.79 |
|  | ΔDBP, mm Hg | -3.44 (-6.20, -0.68) | -0.85 (-2.68, 0.99) | 2.59 (0.14, 5.05) | 0.04 | 0.42 |
| Beta-blockers use (n=379) | | GG (n=75) | GT+TT (n=304) |  |  |  |
|  | ΔSBP, mm Hg | -5.52 (-11.50, 0.47) | -3.95 (-7.91, 0.01) | 1.57 (-4.04, 7.17) | 0.58 | 0.92 |
|  | ΔDBP, mm Hg | -4.96 (-8.08, -1.83) | -4.27 (-6.34, -2.20) | 0.69 (-2.24, 3.62) | 0.65 | 1.00 |

***continued***

| **Variants** | **Changes in BP** | | **Mean (95%CI)* of**  **changes in BP, mm Hg** | | **Mean Difference (95%CI)* of changes in BP, mm Hg** | ***P**** | | **FDR^†^** |
| --- | --- | --- | --- | --- | --- | --- | --- | --- |
| ***PTPRD* rs4742610 C>T** | |  | |  |  | |  |  |
| CCBs use (n=1184) | | CC (n=453) | | CT+TT (n=731) |  | |  |  |
|  | ΔSBP, mm Hg | -4.21 (-6.99, -1.43) | | -2.86 (-5.43, -0.30) | 1.35 (-1.17, 3.86) | | 0.29 | 0.65 |
|  | ΔDBP, mm Hg | -2.97 (-4.41, -1.53) | | -3.22 (-4.55, -1.89) | -0.24 (-1.55, 1.06) | | 0.71 | 0.87 |
| ARBs or ACEIs use (n=1170) | | CC (n=439) | | CT+TT (n=731) |  | |  |  |
|  | ΔSBP, mm Hg | -4.13 (-6.42, -1.85) | | -3.75 (-5.71, -1.79) | 0.39 (-2.05, 2.82) | | 0.76 | 0.76 |
|  | ΔDBP, mm Hg | -2.85 (-4.04, -1.65) | | -3.12 (-4.15, -2.09) | -0.27 (-1.55, 1.00) | | 0.67 | 0.82 |
| Diuretics use (n=414) | | CC (n=161) | | CT+TT (n=253) |  | |  |  |
|  | ΔSBP, mm Hg | -4.61 (-9.09, -0.14) | | -1.74 (-5.73, 2.26) | 2.88 (-1.39, 7.15) | | 0.19 | 0.69 |
|  | ΔDBP, mm Hg | -2.09 (-4.32, 0.13) | | -0.68 (-2.67, 1.30) | 1.41 (-0.71, 3.54) | | 0.19 | 0.53 |
| Beta-blockers use (n=380) | | CC (n=151) | | CT+TT (n=229) |  | |  |  |
|  | ΔSBP, mm Hg | -6.67 (-11.36, -1.98) | | -2.74 (-6.95, 1.48) | 3.93 (-0.61, 8.47) | | 0.09 | 0.99 |
|  | ΔDBP, mm Hg | -4.13 (-6.59, -1.67) | | -4.49 (-6.70, -2.28) | -0.37 (-2.75, 2.01) | | 0.76 | 1.00 |
| ***NR1H3* rs11039149 A>G** | |  | |  |  | |  |  |
| CCBs use (n=1184) | | AA (n=1112) | | AG (n=72) |  | |  |  |
|  | ΔSBP, mm Hg | -2.49 (-4.45, -0.53) | | 5.55 (0.64, 10.46) | 8.04 (3.28, 12.81) | | 0.001 | 0.01 |
|  | ΔDBP, mm Hg | -3.21 (-4.43, -1.98) | | -1.52 (-4.30, 1.26) | 1.69 (-0.95, 4.33) | | 0.21 | 0.58 |
| ARBs or ACEIs use (n=1170) | | AA (n=1103) | | AG (n=67) |  | |  |  |
|  | ΔSBP, mm Hg | -4.05 (-5.79, -2.30) | | -1.12 (-6.22, 3.98) | 2.93 (-2.13, 7.99) | | 0.26 | 0.94 |
|  | ΔDBP, mm Hg | -3.16 (-4.08, -2.25) | | -0.26 (-2.93, 2.41) | 2.91 (0.26, 5.56) | | 0.03 | 0.34 |
| Diuretics use (n=414) | | AA (n=390) | | AG (n=24) |  | |  |  |
|  | ΔSBP, mm Hg | -3.08 (-6.73, 0.57) | | 2.55 (-6.59, 11.69) | 5.63 (-3.12, 14.38) | | 0.21 | 0.57 |
|  | ΔDBP, mm Hg | -1.36 (-3.17, 0.46) | | 1.73 (-2.82, 6.27) | 3.08 (-1.26, 7.43) | | 0.17 | 0.91 |
| Beta-blockers use (n=380) | | AA (n=360) | | AG (n=20) |  | |  |  |
|  | ΔSBP, mm Hg | -4.10 (-7.96, -0.24) | | -7.76 (-17.96, 2.43) | -3.66 (-13.64, 6.32) | | 0.47 | 0.87 |
|  | ΔDBP, mm Hg | -4.24 (-6.26, -2.23) | | -6.20 (-11.53, -0.88) | -1.96 (-7.17, 3.26) | | 0.46 | 1.00 |
| ***MMP3* rs32025058 A>del** | |  | |  |  | |  |  |
| CCBs use (n=1181) | | AA (n=832) | | A/- + -/- (n=349) |  | |  |  |
|  | ΔSBP, mm Hg | -3.56 (-6.05, -1.08) | | -3.33 (-6.31, -0.34) | 0.24 (-2.43, 2.91) | | 0.86 | 0.95 |
|  | ΔDBP, mm Hg | -3.31 (-4.60, -2.02) | | -2.81 (-4.36, -1.26) | 0.50 (-0.88, 1.89) | | 0.48 | 0.87 |
| ARBs or ACEIs use (n=1168) | | AA (n=785) | | A/- + -/- (n=383) |  | |  |  |
|  | ΔSBP, mm Hg | -4.10 (-5.97, -2.22) | | -3.58 (-6.06, -1.10) | 0.52 (-1.99, 3.03) | | 0.69 | 0.84 |
|  | ΔDBP, mm Hg | -3.08 (-4.06, -2.10) | | -2.93 (-4.23, -1.62) | 0.15 (-1.16, 1.47) | | 0.82 | 0.90 |
| Diuretics use (n=413) | | AA (n=294) | | A/- + -/- (n=119) |  | |  |  |
|  | ΔSBP, mm Hg | -3.41 (-7.26, 0.43) | | -1.22 (-6.12, 3.69) | 2.20 (-2.33, 6.72) | | 0.34 | 0.75 |
|  | ΔDBP, mm Hg | -1.34 (-3.26, 0.57) | | -0.87 (-3.31, 1.57) | 0.47 (-1.79, 2.72) | | 0.68 | 0.68 |
| Beta-blockers use (n=378) | | AA (n=275) | | A/- + -/- (n=103) |  | |  |  |
|  | ΔSBP, mm Hg | -4.53 (-8.52, -0.54) | | -3.52 (-8.87, 1.84) | 1.01 (-3.98, 6.01) | | 0.69 | 0.84 |
|  | ΔDBP, mm Hg | -4.25 (-6.35, -2.15) | | -4.88 (-7.70, -2.06) | -0.63 (-3.26, 1.99) | | 0.64 | 1.00 |
| ***CACNA1C* rs1051375 A>G** | |  | |  |  | |  |  |
| CCBs use (n=1177) | | AA (n=519) | | AG+GG (n=658) |  | |  |  |
|  | ΔSBP, mm Hg | -4.62 (-7.36, -1.88) | | -2.48 (-5.09, 0.13) | 2.14 (-0.32, 4.60) | | 0.09 | 0.33 |
|  | ΔDBP, mm Hg | -3.55 (-4.96, -2.13) | | -2.65 (-3.99, -1.30) | 0.90 (-0.37, 2.18) | | 0.17 | 1.00 |
| ARBs or ACEIs use (n=1160) | | AA (n=508) | | AG+GG (n=652) |  | |  |  |
|  | ΔSBP, mm Hg | -4.72 (-6.90, -2.54) | | -3.34 (-5.36, -1.32) | 1.38 (-1.00, 3.76) | | 0.26 | 1.00 |
|  | ΔDBP, mm Hg | -3.44 (-4.59, -2.30) | | -2.6 (-3.66, -1.54) | 0.85 (-0.40, 2.09) | | 0.18 | 0.50 |
| Diuretics use (n=413) | | AA (n=182) | | AG+GG (n=231) |  | |  |  |
|  | ΔSBP, mm Hg | -5.50 (-9.77, -1.22) | | -0.64 (-4.69, 3.40) | 4.85 (0.76, 8.95) | | 0.02 | 0.22 |
|  | ΔDBP, mm Hg | -1.79 (-3.93, 0.35) | | -0.75 (-2.78, 1.27) | 1.04 (-1.01, 3.09) | | 0.32 | 0.51 |
| Beta-blockers use (n=379) | | AA (n=162) | | AG+GG (n=217) |  | |  |  |
|  | ΔSBP, mm Hg | -6.41 (-11.07, -1.74) | | -2.86 (-7.11, 1.38) | 3.54 (-0.99, 8.08) | | 0.13 | 0.69 |
|  | ΔDBP, mm Hg | -4.49 (-6.94, -2.05) | | -4.23 (-6.45, -2.00) | 0.26 (-2.11, 2.64) | | 0.83 | 1.00 |

***continued***

| **Variants** | | **Changes in BP** | | **Mean (95%CI)* of**  **changes in BP, mm Hg** | | **Mean Difference (95%CI)* of changes in BP, mm Hg** | ***P**** | | **FDR^†^** | |
| --- | --- | --- | --- | --- | --- | --- | --- | --- | --- | --- |
| ***NEDD4L* rs4149601 G>A** | | |  | |  |  | |  | |  |
| CCBs use (n=1181) | | | GG (n=829) | | GA+AA (n=352) |  | |  | |  |
|  | ΔSBP, mm Hg | | -2.89 (-5.38, -0.40) | | -4.48 (-7.48, -1.49) | -1.59 (-4.25, 1.07) | | 0.24 | | 0.66 |
|  | ΔDBP, mm Hg | | -3.03 (-4.32, -1.74) | | -3.26 (-4.81, -1.71) | -0.24 (-1.61, 1.14) | | 0.74 | | 0.81 |
| ARBs or ACEIs use (n=1167) | | | GG (n=821) | | GA+AA (n=346) |  | |  | |  |
|  | ΔSBP, mm Hg | | -3.48 (-5.36, -1.59) | | -4.96 (-7.46, -2.45) | -1.48 (-4.05, 1.09) | | 0.26 | | 0.71 |
|  | ΔDBP, mm Hg | | -2.99 (-3.98, -2.01) | | -2.96 (-4.27, -1.65) | 0.03 (-1.31, 1.38) | | 0.96 | | 0.96 |
| Diuretics use (n=414) | | | GG (n=284) | | GA+AA (n=130) |  | |  | |  |
|  | ΔSBP, mm Hg | | -3.35 (-7.20, 0.50) | | -1.63 (-6.43, 3.17) | 1.72 (-2.67, 6.12) | | 0.44 | | 0.70 |
|  | ΔDBP, mm Hg | | -1.62 (-3.53, 0.29) | | -0.27 (-2.65, 2.11) | 1.35 (-0.84, 3.53) | | 0.23 | | 0.50 |
| Beta-blockers use (n=380) | | | GG (n=269) | | GA+AA (n=111) |  | |  | |  |
|  | ΔSBP, mm Hg | | -3.90 (-7.99, 0.18) | | -5.24 (-10.4, -0.08) | -1.34 (-6.24, 3.57) | | 0.59 | | 0.82 |
|  | ΔDBP, mm Hg | | -4.42 (-6.55, -2.28) | | -4.19 (-6.88, -1.49) | 0.23 (-2.33, 2.80) | | 0.86 | | 1.00 |
| ***NEDD4L* rs292449 C>G** | | |  | |  |  | |  | |  |
| CCBs use (n=1183) | | | CC (n=749) | | CG+GG (n=434) |  | |  | |  |
|  | ΔSBP, mm Hg | | -3.57 (-6.10, -1.05) | | -3.08 (-5.94, -0.23) | 0.49 (-2.04, 3.01) | | 0.70 | | 1.00 |
|  | ΔDBP, mm Hg | | -3.11 (-4.41, -1.80) | | -3.11 (-4.59, -1.63) | -0.01 (-1.31, 1.30) | | 0.99 | | 0.99 |
| ARBs or ACEIs use (n=1170) | | | CC (n=745) | | CG+GG (n=425) |  | |  | |  |
|  | ΔSBP, mm Hg | | -3.52 (-5.45, -1.59) | | -4.56 (-6.89, -2.24) | -1.04 (-3.48, 1.40) | | 0.40 | | 0.74 |
|  | ΔDBP, mm Hg | | -2.60 (-3.61, -1.59) | | -3.76 (-4.97, -2.54) | -1.16 (-2.43, 0.12) | | 0.08 | | 0.42 |
| Diuretics use (n=414) | | | CC (n=262) | | CG+GG (n=152) |  | |  | |  |
|  | ΔSBP, mm Hg | | -1.73 (-5.66, 2.21) | | -4.89 (-9.44, -0.33) | -3.16 (-7.43, 1.11) | | 0.15 | | 0.81 |
|  | ΔDBP, mm Hg | | -0.90 (-2.86, 1.06) | | -1.84 (-4.10, 0.43) | -0.94 (-3.07, 1.19) | | 0.39 | | 0.42 |
| Beta-blockers use (n=380) | | | CC (n=234) | | CG+GG (n=146) |  | |  | |  |
|  | ΔSBP, mm Hg | | -4.52 (-8.63, -0.41) | | -3.83 (-8.78, 1.13) | 0.70 (-3.97, 5.36) | | 0.77 | | 0.85 |
|  | ΔDBP, mm Hg | | -4.28 (-6.43, -2.13) | | -4.49 (-7.08, -1.90) | -0.21 (-2.65, 2.22) | | 0.86 | | 1.00 |
| ***ACE2* rs2106809 G>A** | | |  | |  |  | |  | |  |
| CCBs use (n=1184) | | | GG (n=472) | | GA+AA (n=712) |  | |  | |  |
|  | ΔSBP, mm Hg | | -2.22 (-4.93, 0.50) | | -4.46 (-7.10, -1.83) | -2.25 (-4.80, 0.31) | | 0.09 | | 0.47 |
|  | ΔDBP, mm Hg | | -2.98 (-4.39, -1.57) | | -3.24 (-4.60, -1.87) | -0.26 (-1.59, 1.07) | | 0.70 | | 0.97 |
| ARBs or ACEIs use (n=1169) | | | GG (n=484) | | GA+AA (n=685) |  | |  | |  |
|  | ΔSBP, mm Hg | | -3.28 (-5.45, -1.10) | | -4.46 (-6.51, -2.41) | -1.18 (-3.62, 1.25) | | 0.34 | | 0.75 |
|  | ΔDBP, mm Hg | | -3.19 (-4.33, -2.05) | | -2.88 (-3.95, -1.80) | 0.31 (-0.96, 1.59) | | 0.63 | | 0.87 |
| Diuretics use (n=414) | | | GG (n=166) | | GA+AA (n=248) |  | |  | |  |
|  | ΔSBP, mm Hg | | -2.32 (-6.64, 1.99) | | -3.32 (-7.50, 0.85) | -1.00 (-5.36, 3.36) | | 0.65 | | 0.90 |
|  | ΔDBP, mm Hg | | -2.01 (-4.15, 0.13) | | -0.54 (-2.62, 1.53) | 1.46 (-0.70, 3.63) | | 0.19 | | 0.68 |
| Beta-blockers use (n=380) | | | GG (n=167) | | GA+AA (n=213) |  | |  | |  |
|  | ΔSBP, mm Hg | | -3.96 (-8.50, 0.58) | | -4.60 (-9.02, -0.18) | -0.64 (-5.30, 4.03) | | 0.79 | | 0.79 |
|  | ΔDBP, mm Hg | | -4.02 (-6.39, -1.65) | | -4.65 (-6.96, -2.34) | -0.63 (-3.07, 1.80) | | 0.61 | | 1.00 |

Abbreviations: BP, blood pressure; CI, confidence interval; FDR, false discovery rate; CCB, calcium channel blocker; ARB, angiotensin receptor blocker; ACEI, angiotensin converting enzyme inhibitor; ΔSBP, changes in systolic blood pressure; ΔDBP, changes in diastolic blood pressure.

*Means, mean differences, 95%CIs and *P*-values were calculated by generalized linear regression model with adjustment for baseline characteristics including age, sex, body mass index, serum creatine, blood pressure, alcohol intake, smoke status and antihypertensive drugs. Mean differences were calculated between Mm+mm and MM genotypes.

†FDRs were calculated by the Benjamin & Hochberg method.

The full names of the genes are the same as mentioned in the footnote of Supplementary Table S1.

**Supplementary Table S12. Sensitivity analysis on association of *NR1H3* variant rs11039149 A>G with SBP response to CCBs therapy after excluding 100 patients with irregular drug taking (n=1737).**

| **Genotype** | **Mean (95%CI)***  **of ΔSBP, mm Hg** | **Mean Difference (95%CI)***  **of ΔSBP, mm Hg** | ***P**** |
| --- | --- | --- | --- |
| ***NR1H3* rs11039149 A>G** | |  |  |
| CCBs therapy (n=1129) | |  |  |
| **AA (n=1060)** | -2.17 (-4.16, -0.18) | 6.81 (1.95, 11.68) | 0.006 |
| **AG (n=69)** | 4.64 (-0.36, 9.65) |  |  |
| CCBs monotherapy (n=324) | |  |  |
| **AA (n=302)** | -1.93 (-7.93, 4.07) | 14.02 (4.90, 23.14) | 0.003 |
| **AG (n=22)** | 12.09 (1.69, 22.49) |  |  |
| CCBs multitherapy (n=805) | |  |  |
| **AA (n=758)** | -3.14 (-5.58, -0.70) | 3.44 (-2.80, 9.68) | 0.15 |
| **AG (n=47)** | 0.29 (-6.09, 6.67) |  |  |
| non CCBs therapy (n=608) | |  |  |
| **AA (n=571)** | -3.40 (-5.55, -1.25) | 2.29 (-4.35, 8.92) | 0.50 |
| **AG (n=37)** | -1.11 (-7.70, 5.48) |  |  |

Abbreviations: *NR1H3*, nuclear receptor subfamily 1 group H member 3; SBP, systolic blood pressure; CCBs, calcium channel blockers; CI, confidence interval; ΔSBP, changes in systolic blood pressure.

*Means, mean differences, 95%CIs and *P*-values were calculated by generalized linear regression model with adjustment for baseline characteristics including age, sex, body mass index, serum creatine, blood pressure, alcohol intake, smoke status and antihypertensive drugs. Mean differences were calculated between AG and AA genotypes.

**Supplementary Table S13. Sensitivity analysis on association of *NR1H3* variant rs11039149 A>G with SBP response to CCBs therapy after excluding 531 patients with change in drug regimen (n=1306).**

| **Genotype** | **Mean (95%CI)***  **of ΔSBP, mm Hg** | **Mean Difference (95%CI)***  **of ΔSBP, mm Hg** | ***P**** |
| --- | --- | --- | --- |
| ***NR1H3* rs11039149 A>G** | |  |  |
| CCBs therapy (n=892) | |  |  |
| **AA (n=843)** | -3.99 (-6.35, -1.62) | 10.65 (4.91, 16.39) | <0.001 |
| **AG (n=49)** | 6.66 (0.70, 12.63) |  |  |
| CCBs monotherapy (n=286) | |  |  |
| **AA (n=267)** | -3.77 (-10.95, 3.41) | 19.51 (9.79, 29.23) | <0.001 |
| **AG (n=19)** | 15.74 (4.17, 27.31) |  |  |
| CCBs multitherapy (n=606) | |  |  |
| **AA (n=576)** | -4.58 (-7.45, -1.70) | 5.44 (-2.27, 13.16) | 0.17 |
| **AG (n=30)** | 0.86 (-7.04, 8.77) |  |  |
| non CCBs therapy (n=414) | |  |  |
| **AA (n=391)** | -3.62 (-6.21, -1.04) | 3.45 (-4.94, 11.83) | 0.42 |
| **AG (n=23)** | -0.18 (-8.47, 8.11) |  |  |

Abbreviations: *NR1H3*, nuclear receptor subfamily 1 group H member 3; SBP, systolic blood pressure; CCBs, calcium channel blockers; CI, confidence interval; ΔSBP, changes in systolic blood pressure.

*Means, mean differences, 95%CIs and *P*-values were calculated by generalized linear regression model with adjustment for baseline characteristics including age, sex, body mass index, serum creatine, blood pressure, alcohol intake, smoke status and antihypertensive drugs. Mean differences were calculated between AG and AA genotypes.

**Supplementary Table S14. Association of *NR1H3* variant rs11039149 A>G with SBP response to CCBs therapy calculated by multiple linear mixing model.**

| **Genotype** | **Mean (95%CI)***  **of ΔSBP, mm Hg** | **Mean Difference (95%CI)* of ΔSBP, mm Hg** | ***P**** |
| --- | --- | --- | --- |
| ***NR1H3* rs11039149 A>G** | |  |  |
| CCBs therapy (n=1184) | |  |  |
| **AA (n=1112)** | -3.53 (-5.93, -1.14) | 7.29 (2.15, 12.43) | 0.007 |
| **AG (n=72)** | 3.76 (1.63, 9.75) |  |  |
| CCBs monotherapy (n=359) | |  |  |
| **AA (n=335)** | -5.71 (-7.59, 3.82) | 14.70 (7.43, 21.97) | 0.001 |
| **AG (n=24)** | 8.99 (1.96, 16.01) |  |  |
| CCBs multitherapy (n=825) | |  |  |
| **AA (n=777)** | -4.70 (-7.30, -2.10) | 3.46 (-3.21, 10.13) | 0.29 |
| **AG (n=48)** | 1.24 (-8.05, 5.57) |  |  |
| non CCBs therapy (n=653) | |  |  |
| **AA (n=614)** | 0.57 (-1.87, 3.00) | 2.10 (-3.45, 7.64) | 0.46 |
| **AG (n=39)** | 2.66 (-3.16, 8.48) |  |  |

Abbreviations: *NR1H3*, nuclear receptor subfamily 1 group H member 3; SBP, systolic blood pressure; CCBs, calcium channel blockers; CI, confidence interval; ΔSBP, changes in systolic blood pressure.

*Means, mean differences, 95%CIs and *P*-values were calculated by multiple linear mixing model. Baseline characteristics of age, sex, body mass index, serum creatine, blood pressure, alcohol intake, smoke status and antihypertensive drugs were adjusted as fixed effect, entry time and center were adjusted as random effect. Mean differences were calculated between AG and AA genotypes.

**Supplementary Table S15. Survival analysis between variants and cardiovascular events.**

| **Variants** | **Person-years** | **CVD events, n (%)** | **Crude HR** | ***P**** | **FDR^†^** | **Adjusted HR** | ***P*^#^** | **FDR^†^** |
| --- | --- | --- | --- | --- | --- | --- | --- | --- |
|  |  |  | **(95%CI)*** |  |  | **(95%CI)^#^** |  |  |
| ***AGT* (rs699 G>A)** | |  |  |  |  |  |  |  |
| GG (n=1181) | 3637.7 | 98 (8.3%) | 1.00 |  |  | 1.00 |  |  |
| GA+AA (n=648) | 2028.1 | 60 (9.3%) | 1.07 (0.77-1.49) | 0.69 | 0.84 | 1.12 (0.80-1.57) | 0.49 | 1.00 |
| ***AGT* (rs5051 T>C)** | |  |  |  |  |  |  |  |
| TT (n=1180) | 3633.2 | 98 (8.3%) | 1.00 |  |  | 1.00 |  |  |
| TC+CC (n=652) | 2042.3 | 61 (9.4%) | 1.08 (0.78-1.50) | 0.65 | 1.00 | 1.13 (0.81-1.58) | 0.81 | 1.00 |
| ***AGTR1* (rs5186 A>C)** | |  |  |  |  |  |  |  |
| AA (n=1646) | 5093.7 | 134 (8.1%) | 1.00 |  |  | 1.00 |  |  |
| AC+CC (n=191) | 603.4 | 25 (13.1%) | 1.60 (1.04-2.48) | 0.04 | 0.44 | 1.67 (1.07-2.59) | 0.02 | 0.22 |
| ***ADD1* (rs4961 G>T)** | |  |  |  |  |  |  |  |
| GG (n=416) | 1324.5 | 32 (7.7%) | 1.00 |  |  | 1.00 |  |  |
| TG+TT (n=1418) | 4361.2 | 127 (9.0%) | 1.16 (0.78-1.72) | 0.48 | 0.88 | 1.04 (0.70-1.56) | 0.83 | 1.00 |
| ***PTPRD* (rs4742610 C>T)** | |  |  |  |  |  |  |  |
| CC (n=693) | 2102.1 | 67 (9.7%) | 1.00 |  |  | 1.00 |  |  |
| CT+TT (n=1144) | 3595.3 | 92 (8.0%) | 0.80 (0.57-1.10) | 0.17 | 0.62 | 0.86 (0.62-1.19) | 0.35 | 1.00 |
| ***NR1H3* (rs11039149 A>G)** | |  |  |  |  |  |  |  |
| AA (n=1726) | 5345.6 | 155 (9.0%) | 1.00 |  |  | 1.00 |  |  |
| AG (n=111) | 351.4 | 4 (3.6%) | 0.42 (0.15-1.12) | 0.08 | 0.44 | 0.40 (0.15-1.10) | 0.08 | 0.44 |
| ***MMP3* (rs32025058 A>del)** | |  |  |  |  |  |  |  |
| AA (n=1269) | 4017.1 | 110 (8.7%) | 1.00 |  |  | 1.00 |  |  |
| -/- (n=563) | 1652.2 | 49 (8.7%) | 1.14 (0.80-1.61) | 0.48 | 1.00 | 1.10 (0.78-1.57) | 0.59 | 1.00 |
| ***CACNA1C* (rs1051375 A>G)** | |  |  |  |  |  |  |  |
| AA (n=795) | 2468.6 | 66 (8.3%) | 1.00 |  |  | 1.00 |  |  |
| AG+GG (n=1028) | 3186.2 | 91 (8.9%) | 1.03 (0.74-1.43) | 0.86 | 0.95 | 1.08 (0.77-1.50) | 0.66 | 1.00 |
| ***NEDD4L* (rs4149601 G>A)** | |  |  |  |  |  |  |  |
| GG (n=1296) | 4022.5 | 114 (8.8%) | 1.00 |  |  | 1.00 |  |  |
| GA+AA (n=537) | 1655.3 | 45 (8.4%) | 0.87 (0.61-1.26) | 0.47 | 1.00 | 0.90 (0.63-1.31) | 0.59 | 1.00 |
| ***NEDD4L* (rs292449 C>G)** | |  |  |  |  |  |  |  |
| CC (n=1171) | 3647.5 | 102 (8.7%) | 1.00 |  |  | 1.00 |  |  |
| CG+GG (n=665) | 2053.1 | 57 (8.6%) | 0.99 (0.71-1.38) | 0.95 | 0.95 | 1.00 (0.72-1.41) | 0.98 | 0.98 |
| ***ACE2* (rs2106809 G>A)** | |  |  |  |  |  |  |  |
| GG (n=733) | 2290.6 | 67 (9.1%) | 1.00 |  |  | 1.00 |  |  |
| GA+AA (n=1103) | 3406.1 | 91 (8.3%) | 0.93 (0.67-1.29) | 0.67 | 0.92 | 1.04 (0.74-1.45) | 0.84 | 0.93 |

Abbreviations: CVD, cardiovascular disease; HR, hazards ratio; CI, confidence interval; FDR, false discovery rate.

*Crude HRs (95%CIs) and *P*-values were obtained with Cox regression model.

#Adjusted HRs (95%CIs) and *P*-values were obtained with multivariate Cox regression analysis with adjustment for baseline characteristics including age, sex, body mass index, serum creatine, blood pressure, alcohol intake, smoke status and antihypertensive drugs.

†FDRs were calculated by the Benjamin & Hochberg method.

The full names of the genes are the same as mentioned in the footnote of Supplementary Table S1.
